# Supplementary material for: Awakening a latent carbon fixation cycle in Escherichia coli
Source: Nat Commun. 2020 Nov 16;11:5812. doi: 10.1038/s41467-020-19564-5 (PMC7669889; doi:10.1038/s41467-020-19564-5)
Supplement: Supplementary file 1 — Supplementary Information [file 41467_2020_19564_MOESM1_ESM.pdf]

Awakening a latent carbon fixation cycle in *Escherichia coli*

Satanowski and Dronsella *et al.*

Supplementary Method 1. General description of MILP-based algorithm for metabolic pathway design

We use a similar approach to the OptMDFpathway method [1]. We set up a Mixed Integer Linear Problem (MILP)-based optimization problem which simultaneously looks for solutions that balance an objective reaction (here, 3 CO_2 → pyruvate), and maximize the Max-min Driving Force (MDF) [3] while simultaneously minimizing the number of reactions. In the section titled 'Alterations to the iML1515 model', we lay out the changes we made to the set of reactions in the iML1515 model [2]. In the section titled 'Formulation of the Mixed Integer Linear Problem', we describe how the MILP problem is formulated. In the section titled 'Iterating the space of solutions', we describe how we use the MILP framework to find also sub-optimal solutions and cover a large part of the feasible solution space of carbon fixation pathways.

We made a few changes to the genome-scale model of *E. coli* (iML1515) [2]:

- Removing all non-cytoplasmic reactions (i.e. exchange or transport reactions), except for exchange reactions of inorganic metabolites: protons, water, orthophosphate, ammonium, and oxygen
- Removing all boundary reactions (i.e. sink reactions needed to allow certain co-factors to leave the system)
- Adding co-factor regenerating reactions: ADP → ATP, $NADP^+$ → NADPH, and NAD^+ → NADH
- Replacing all flavoredoxins and thioredoxins with NADP(H): We replaced all flavoredoxins and thioredoxins with NADP(H), since we do not have a good estimate of their reduction potential, and therefore the MDF for pathways using them was artificially high. We can assume that the electrons used for reducing CO_2 in the carbon fixation cycle ultimately have to pass through NADPH, and therefore a simple solution was to replace the electron donor with NADPH. This way, we could keep the flavoredoxins/thioredoxins-dependent reactions in the model while having a more realistic estimate of their thermodynamics.
- Removing the formate-tetrahydrofolate ligase reaction (FTHFLi): We found that the reaction formate-tetrahydrofolate ligase (FTHFLi) appears in some of the solutions although the gene associated with this reaction is unknown. FTHFLi was thus excluded from our model altogether. Notably, removing this reaction does not significantly affect the space of solutions, because it can be easily replaced by GAR transformylase-T (GART) and the reverse reaction of Phosphoribosylglycinamide formyltransferase (GARFT).
- Add the objective reaction (OBJ): 3 CO_2 → pyruvate
- Setting the bounds (the range of possible fluxes) of all remaining reactions to be between -10 and 10

The mixed integer linear problem was formulated as follows:

$$\text{Maximize } B - \sum_i z_i \quad (1)$$

Such that:

$$Sv = 0 \quad (2)$$
$$v_{OBJ} = -1 \quad (3)$$
$$v - \beta z \leq 0 \quad (4)$$
$$B \leq -g^0 - S^\top x + M(1 - z) \quad (5)$$
$$z \in \{0,1\}^n \quad (6)$$
$$0 \leq v \leq \beta \quad (7)$$
$$ln\,(C_{min}) \leq x \leq ln\,(C_{max}) \quad (8)$$
$$0 \leq B \quad (9)$$

where the vector *v* contains the relative reaction rates, *z* are the Boolean reaction indicators, *x* are the log-scaled metabolite concentrations, and g^0 is a vector of all the reactions' standard Gibbs free energies in units of *RT* (see Equation **10**).

$$\forall i\ g_i{}^0 \equiv \Delta_r G_i{}'^0 / RT \quad (10)$$

β is a parameter that limits the maximal rate for each single reaction in the pathway (relative to the objective reaction, i.e. 3 CO_2 → pyruvate), and was set arbitrarily to 10. The rate of the objective reaction (v_{OBJ}) is set to be exactly -1 (Equation **3**). This ensures that any pathway solution would exactly balance it, i.e. the overall reaction in the pathway would be 3 CO_2 → pyruvate. *M* is a parameter which has a large value, much higher than any of the values in g^0. The lower and upper bounds on the concentrations of most metabolites were set to 1 μM and 10 mM. Only 14 central metabolites and co-factors were confined to more specific ranges based on physiological data (see Supplementary Table 3).

Note that as a pre-processing step, we split all reactions to a forward and backward reaction and therefore all (uni-directional) rates must be positive. Equation **4** ensures that each reaction indicator (z_i) can be equal to 0, only if the rate is 0. We don't need to care about it being equal to 1 even if a reaction is not active, since the optimization goal (which maximizes the sum of all indicators) will prevent that.

Equation **5** ensures that every active reaction has a positive driving force (which is given by $-g_i{}^0 - \sum_j S_{ij} x_j$). If $z_i = 1$, the driving force must be larger than *B*, which is a positive number that represents a margin. We add *B* to the optimization function, in order to maximize this margin. This approach is based on the Max-min Driving Force [3] and aims to prioritize pathways that can be operated as far from equilibrium as possible. The method for using Max-min Driving Force optimization for finding feasible pathways in the genome-scale *E. coli* model was introduced by Hädicke *et al*. [1], and denoted OptMDFpathway.

Our MILP objective function is the margin (*B*) minus the sum of all indicators (which is equal to the number of reactions in the pathway). Maximizing this function will simultaneously maximize the MDF and minimize the pathway length. Importantly, when combining two optimization functions, the relative weight given to each one is very important. Since the MDF is given in units of *RT*, and the pathway length is an integer, using equal weights is an arbitrary choice. For example, giving a much higher weight to the MDF (by changing the units, or multiplying it by a large pre-factor) would likely change the MILP solution. In this work, we wanted to avoid tuning the relative optimization weights. Instead, we iterate the space of sub-optimal solutions and try to identify pathways that are Pareto-optimal (i.e., no other solution outperforms them in both MDF and length). This procedure is explained in detail in the next section.

In order to cover the space of thermodynamically feasible solutions (i.e. pathways with MDF > 0), we iteratively use integer-cuts to eliminate all previous solutions and find the next optimal one [4]. Formally, if P_0 , … , P_m are the set of solutions already discovered by our algorithm (where $P_j \subset \{0, \ldots, n\}$) then the added constraints will be:

$$\forall j \sum_{i \in P_j} z_i < |P_j| \tag{11}$$

where $|P_j|$ is the size of the pathway (i.e. the number of reactions). Each one of these constraints eliminates P_j and any pathway which is a superset of P_j from the solution space.

Using the Gurobi solver, we could recover only ~100 solutions per day, on an Intel Core i7-4770S CPU (with 8 cores). However, when running the iterative search for about 3 days, we noticed that even though the solutions were different by at least one reaction, the overlap between them was quite large and running the search exhaustively would take a significant amount of time. Therefore, in order to increase the diversity of the solutions and shorten the run-time, we changed the solution elimination process, so that each time all solutions within a radius of 3 reactions would also be eliminated. To achieve that, we subtract 3 from the right-hand side of each constraint:

$$\forall j \sum_{i \in P_j} z_i < |P_j| - 3 \tag{12}$$

After this modification, the diversity of the pathways within the first 50 was much larger, which we measure by counting which carboxylating enzymes were used in each pathway (see Supplementary Figure 5 and Supplementary Table 4). Since we optimize both the MDF and number of reactions in each iteration, it is very unlikely that pathways that are Pareto-optimal would be excluded from the results due to the 3-radius rule. Nevertheless, we verified that the set of Pareto-optimal solutions is not affected by the exclusion radius.

We find that only 2 pathways are Pareto-optimal in terms of MDF and pathway length. The first one (pathway 0) is the GED cycle, with 17 reactions and an MDF of 3.3 kJ/mol. This is the shortest possible CO_2 fixating pathway in *E. coli*. The only other Pareto-optimal pathway, comprising 20 reactions and an MDF of 5.1 kJ/mol, uses the reverse glycine cleavage system as its carboxylating mechanism.

Graphical depictions of these pathways can be found at https://gitlab.com/elad.noor/ged-cycle/-/tree/master/gdot.

The source code and all input and output files can be found at GitLab (https://gitlab.com/elad.noor/ged-cycle) or Zenodo (https://doi.org/10.5281/zenodo.4066984) under an open-source license (MIT). Specifically, a Jupyter Notebook which was used to create Supplementary Figure 5 and Supplementary Table 4 can be found at https://gitlab.com/elad.noor/ged-cycle/-/blob/master/make_supplementary_figures.ipynb.

Supplementary Table 1: Mutations identified in the evolved ∆PZF (∆pfkAB ∆zwf ∆fsaAB ∆fruK) strains.

Locus / Gene Name	Position (NC_000913.3)	Annotation	Mutation Type	Reference bases	Variant bases	Description/Comments	Ancestral Strain (∆pfkAB ∆zwf ∆fsaAB)	Parental Strain (∆PZF* +pGED)	∆PZF +pGED - Mutant A1	∆PZF +pGED - Mutant A2	∆PZF +pGED - Mutant A3	∆PZF +pGED - Mutant B1	∆PZF +pGED - Mutant B2	∆PZF +pGED - Mutant B3	∆PZF +pGED - Mutant C1	∆PZF +pGED - Mutant C2	∆PZF +pGED - Mutant C3
Cra	88,283	coding region (Q86*)	SNP	C	T	DNA-binding transcriptional dual regulator		+	+	+	+	+	+	+	+	+	+
cydA	771,121	intergenic (-336nt)	IS element insertion	-	-	cytochrome bd-I ubiquinol oxidase subunit I		+	+	+	+	+	+	+	+	+	+
rne	1,142,413	coding region (1954th nt)	Δ2 bp deletion / frameshift	TC	-	ribonuclease E			+	+	+						
dhaM	1,248,151	coding region	IS element insertion	-	-	dihydroxyacetone kinase, phosphotransferase component		+	+	+	+	+	+	+	+	+	+
clsA	1,307,916	coding region (D244N)	SNP	C	T	cardiolipin synthase A			+	+	+						
flhD	1,978,503	intergenic (-400nt)	IS element insertion	-	-	DNA-binding transcriptional dual regulator		+	+	+	+	+	+	+	+	+	+
gatC	2,173,363	coding region	Δ2 bp deletion	CC	-	galactitol-specific PTS enzyme IIC component		+	+	+	+	+	+	+	+	+	+
ptsI	2,535,329	coding region (E422*)	SNP	G	T	phosphoenolpyruvate-protein phosphotransferase PTS enzyme I		+	+	+	+	+	+	+	+	+	+
avtA	3,740,351	coding region (647th nt)	Δ2 bp deletion	TG	-	valine-pyruvate aminotransferase									+		+
avtA	3,740,466	coding region (G254GG)	3bp insertion	-	TGG	valine-pyruvate aminotransferase						+		+			
glpF / zapB	4,118,275	intergenic (-184nt)	IS element insertion	-	-	glycerol facilitator		+	+	+	+	+	+	+	+	+	+
eptA	4,334,799	coding region (silent)	SNP	C	T	phosphoethanolamine transferase		+	+	+	+	+	+	+	+	+	+
leuX (tRNA)**	4,496,406	coding region (2nd nt)	SNP	C	T	tRNA-Leu(CAA)						+	+	+	+	+	+
fimA	4,543,603	coding region (502nd nt)	IS element insertion	-	-	type 1 fimbriae (pili) major subunit							+				
yjjY	4,640,422	coding region	IS element insertion	-	-	protein of unknown function		+	+	+	+	+	+	+	+	+	+
									This strain is referred to as Mutant 'A' in the main text			This strain is referred to as Mutant 'B' in the main text			This strain is referred to as Mutant 'C' in the main text		
									These three colonies were isolated from a single culture evolved for growth on xylose			These three colonies were isolated from a single culture evolved for growth on xylose			These three colonies were isolated from a single culture evolved for growth on xylose		

* abbreviation: ∆PZF = ∆pfkAB ∆zwf ∆fsaAB ∆fruK

** The identified mutation in the L-leucyl-tRNA (leuX) was found in all mutant clones from the independent evolution cultures 'B' and 'C', which display considerably faster growth than mutants from culture 'A'. The mutation is a single-nucleotide polymorphism, located at the second base from the 5'-end of the mature tRNA (i.e. not within its anticodon loop). We hypothesize that this mutation negatively affects maturation and/or folding of the tRNA and thereby modifies translation rates of genes containing UUG-codons (approx. 13% of all leucine-encoding codons), although other leucyl-tRNAs are known to compensate partially for loss-of-function or deletion of leuX (FEBS Letters 344 (1994) 31-34; J. Mol. Biol. (1979) 129, 567-585). Notably, the *edd* gene (encoding 6-phosphogluconate dehydratase, one of the key enzymes in the GED pathway) contains 12 codons normally recognized by leuX. Therefore it is possible that a loss-of-function in leuX optimized expression of *edd* at the translational level, possibly by reducing its protein abundance or by improving *edd* maturation (e.g. incorporation of the required iron-sulfur cluster) via slowed translation.

Note: Plus symbols indicate the presence of an identified mutation.

Supplementary Table 2: List of DNA oligo primers used in this study.

Name	Function	Sequence (5'→3')
rpe-V1	Verification of ∆rpe	GTTTTACGCTGAGTCGCACGTTCCTG
rpe-V2		CGAAGGTCTGGTTGAGCGCCATATTCC
tktA-V1	Verification of ∆tktA	ACAACAAATGTCAATACGCATATCGT
tktA-V2		GTTCCATGTACATGACGCGC
tktB-V1	Verification of ∆tktB	CCACCTTCTCAGACGTTCCC
tktB-V2		GCTTTGACGGTCAGCGTTTT
zwf-V1	Verification of ∆zwf	GCACGAGGCCTGAAAGTGTA
zwf-V2		AAAGCAGTACAGTGCACCGT
sthA-V1	Verification of ∆sthA (∆udhA)	GTTTCTGTTTTGAAGCCGGGGC
sthA-V2		AAACAGACAAAGCAAAGGCCGC
gndA	Amplification of gnd from *E.coli* genomic DNA	GAATGCATCATCACCATCACCACTCCAAGCAACAGATCGGCGTAGTCGG
gndB		GCATCGGTGATTTTTTGCAGGAACTGCGC
gndC		GCGCAGTTCCTGCAAAAAATCACCGATGC
gndD		CGCTAGCTCTAGATTAATCCAGCCATTCGGTATGGAACACACCTTC
edaA	Amplification of eda from *E.coli* genomic DNA	GAATGCATCATCACCATCACCACAAAAACTGGAAAACAAGTGCAGAATCAATCCTGAC CAC
edaB		GAACGGACCCGCAATCGCTTGCAGGGCTTTCAC
edaC		GTGAAAGCCCTGCAAGCGATTGCGGGTCCGTTC
edaD		CGCTAGCTCTAGATTACAGCTTAGCGCCTTCTACAGCTTCACG
eddA	Amplification of edd from *E.coli* genomic DNA	GAATGCATCATCACCATCACCACAATCCACAATTGTTACGCGTAACAAATCGAATCATT GAACG
eddB		CGCTAGCTCTAGATTAAAAAGTGATACAGGTTGCGCCCTGTTCGG
q-rrsA1	qPCR of *rrsA* (reference gene)	CTCTTGCCATCGGATGTGCCCA
q-rrsA2		CCAGTGTGGCTGGTCATCCTCTCA
q-pntA1	qPCR of *pntA*	GCCAATCTGCAACAGTGCTC
q-pntA2		TTTTTGGCTGGATGGCAAGC
CmR-1	Engineering of the pntAB promotor region	TAATACGACTCACTATAGGGCTCCATATGAATATCCTCCTTAG
CmR-2		AATTAACCCTCACTAAAGGGCGGAGCTGCTTCGAAGTTCCTA
PromW-Fwd		GAGCCCTATAGTGAGTCGTATTATCCCTTTGATATTGCATCCCGCGTATATAATATG
PromM-Fwd		GAGCCCTATAGTGAGTCGTATTAACCTATTGACAATTAAAGGCTAAAATGCTATAATTC CAC
PromS-Fwd		GAGCCCTATAGTGAGTCGTATTAAATACTTGACATATCACTGTGATTCACATATAATATG CG
pntA-Prom1		GTACTGGTATTGTTATTAACGAGAAACGTGGCTGATTATTGCATTTAAACAATTAACCCT CACTAAAGGGCG
pntA-Prom2		GCAACACGGGTTTCATTGGTTAACCGTTCTCTTGGTATGCCAATTCGCATTCTTGCCTC TTAACTTTAAAGTTAAACAAAATTATTTCTATTA
pntA-V1	Sequencing of pntA promotor region	AACCCAGTTTCAGCAGCTG
pntA-V2		CCAGGAGGGTGTTCTTAAGC
pfkA-V1	Verification of ∆pfkA	CGTTGGATCACTTCGATGTGC
pfkA-V2		CGAAGCGCATCAGGCATTTT
pfkB-V1	Verification of ∆pfkB	TCTGCAAAATTTTAAATAAAGCTCCAA
pfkB-V2		GAGCATAGTCGGAGAAACGC
fsaA-V1	Verification of ∆fsaA	GCAGCCCAGCGAACAGCCTTTAAG
fsaA-V2		GCTGGCAGGTTATGGCAAACCGG
fsaB-V1	Verification of ∆fsaB	GCCAAACGGCGCAATTTCTACTACC
fsaB-V2		GGGCGATGATGAAGCGCAATTCATC
fruK-V1	Verification of ∆fruK	GGCTTCACTCAGGAACAGCT
fruK-V2		GTTAAGCGCGGAGTTTGTGG

Supplementary Table 3: The allowed concentration ranges for metabolites in the model.

Compound	BiGG identifier	Concentration range
ATP	atp_c	5 mM
ADP	adp_c	0.5 mM - 2.5 mM
AMP	amp_c	0.5 mM - 2.5 mM
NAD+	nad_c	1 mM
NADH	nadh_c	10 μM - 100 μM
NADP+	nadp_c	10 μM
NADPH	nadph_c	10 μM - 100 μM
O2	o2_c	273 μM
CO2	co2_c	6.3 mM
CoA	coa_c	1 mM - 5 mM
orthophosphate	pi_c	1 mM - 10 mM
pyrophosphate	ppi_c	0.5 mM - 1.5 mM
ammonia	nh4_c	1 mM - 10 mM
alpha-ketoglutarate	akg_c	0.5 mM - 5 mM
glutamate	glu__L_c	30 mM - 150 mM

Note: All metabolites that do not appear in this table were constrained by the default ranges of 1 μM to 10 mM.

Supplementary Table 4: The Max-min Driving Force and number of reactions of all 50 top pathways.

carboxylators	Max-min Driving Force (kJ/mol)	Sum of absolute fluxes	Number of unique reactions
GND	3.29	33	17
ME1 + GND	2.93	37	19
GLYCL	5.13	49	20
GND	0.16	35	18
GND	2.19	37	19
GND	1.01	37	19
GND	1.74	39	20
GND	1.4	41	21
ME1 + GND	1.35	37	21
ME1 + GND	0.89	41	21
ME1 + GND	0.89	41	21
GND	0.7	40	22
ME1 + GLYCL	3.12	45	23
ME1 + GND	1.6	44	23
GND	1.42	42	23
GND	1.42	48	23
GND	0.55	45	23
GND	0.46	45	23
GND	0.09	47	23
GND	1.74	47	24
ME1 + GND	1.6	46	24
ME1	1.32	63	24
GND	0.43	47	24
ME1 + GND	1.53	49	25
GND	0.95	58	25
GND	0.9	59	25
ME1 + GND	0.75	46.666	25
GND	0.53	49	25
GND + PPC	2.78	51	26
GND	0.26	44	25
GND + PPC	0.09	46.334	25
GND	0.08	44	25
GND	0.05	46	25
ME1 + GLYCL	3.92	49.334	27
GND	0.75	45.334	26
ME1 + GND	0.75	54	26
GND + PPC	0.75	54	26
GND	0.75	48	26
PPCK	3.12	65.334	27
GND	1.31	48	27
GLYCL + GLXCL	1.23	46	27
GND	0.75	50	27
GND	0.75	50	27
ME1 + GND	0.45	55	27
ME1 + GLYCL	2.87	54	28
ME1 + GLYCL	2.87	55	28
GND	0.16	48	27
GND	0.9	52.334	28
ME1 + GND	0.75	58	28
GND	0.47	58	28

Note: For a graphical depiction of these pathways, see https://gitlab.com/elad.noor/ged-cycle/-/tree/master/gdot.

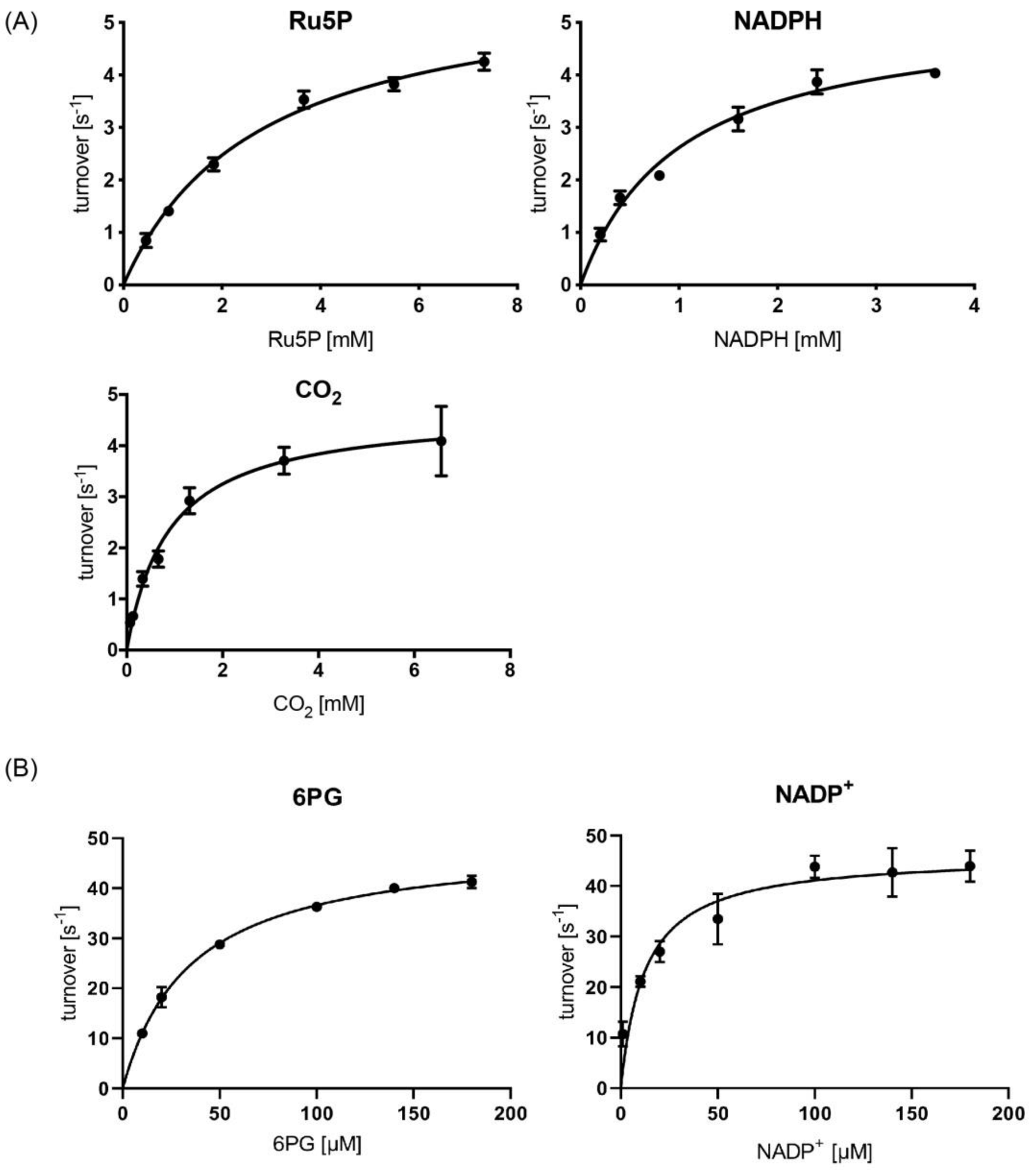

Supplementary Figure 1: Michaelis-Menten kinetics of (A) reductive carboxylation and (B) oxidative decarboxylation by *E.coli* Gnd. Data are presented as mean values +/- SD from three independent experiments (n = 3) and each curve was determined with at least 15 measurements. The data are summarized in the main text. Source data are provided as a Source Data file.

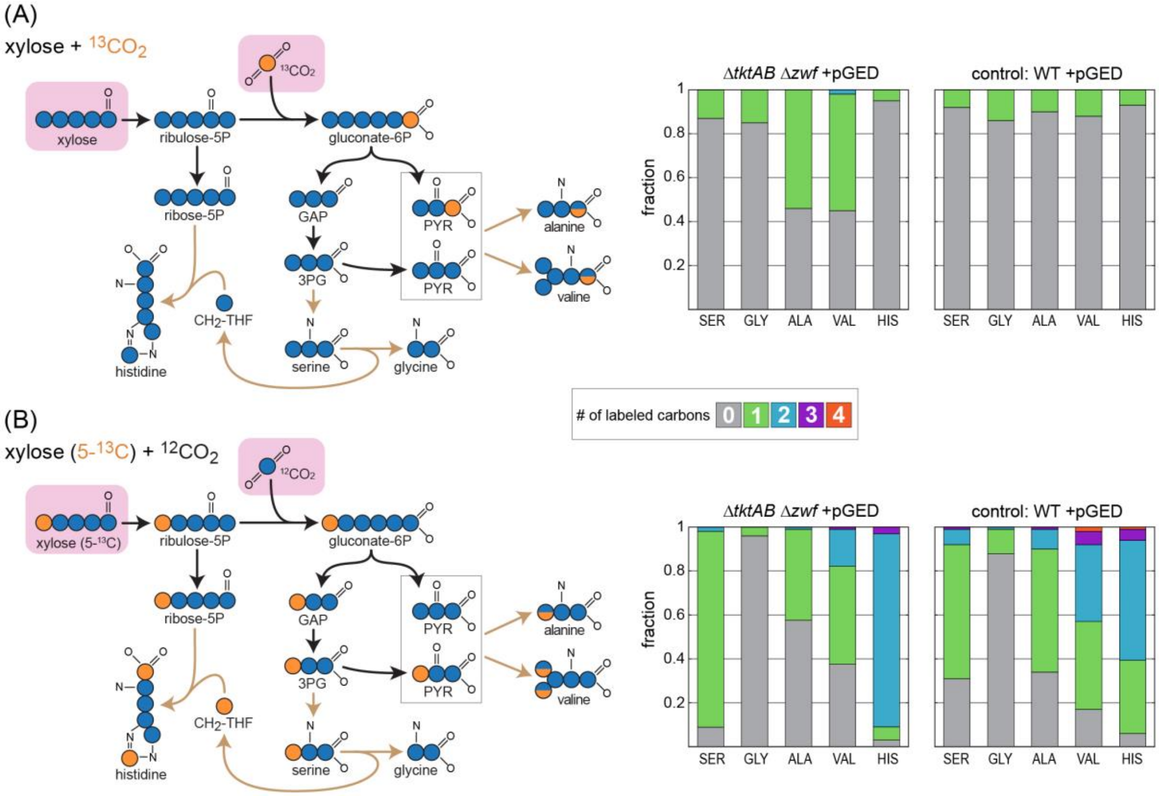

Supplementary Figure 2: ^{13}C-labeling experiments confirm the operation of the GED shunt in the Δ*tktAB* (Δ*zwf*) strain. (A) Cultivation with unlabeled xylose + ^{13}CO2. (B) Cultivation with xylose (5-^{13}C) + unlabeled CO_2. On the left, the expected labeling distributions for the respective feed-stocks are shown. Observed labeling fits the expected pattern and differs from the labeling in a WT strain, which serves as a control. Source data are provided as a Source Data file. Abbreviations: Ala, Alanine; Gly, Glycine; His, Histidine; Ser, Serine; Val, Valine.

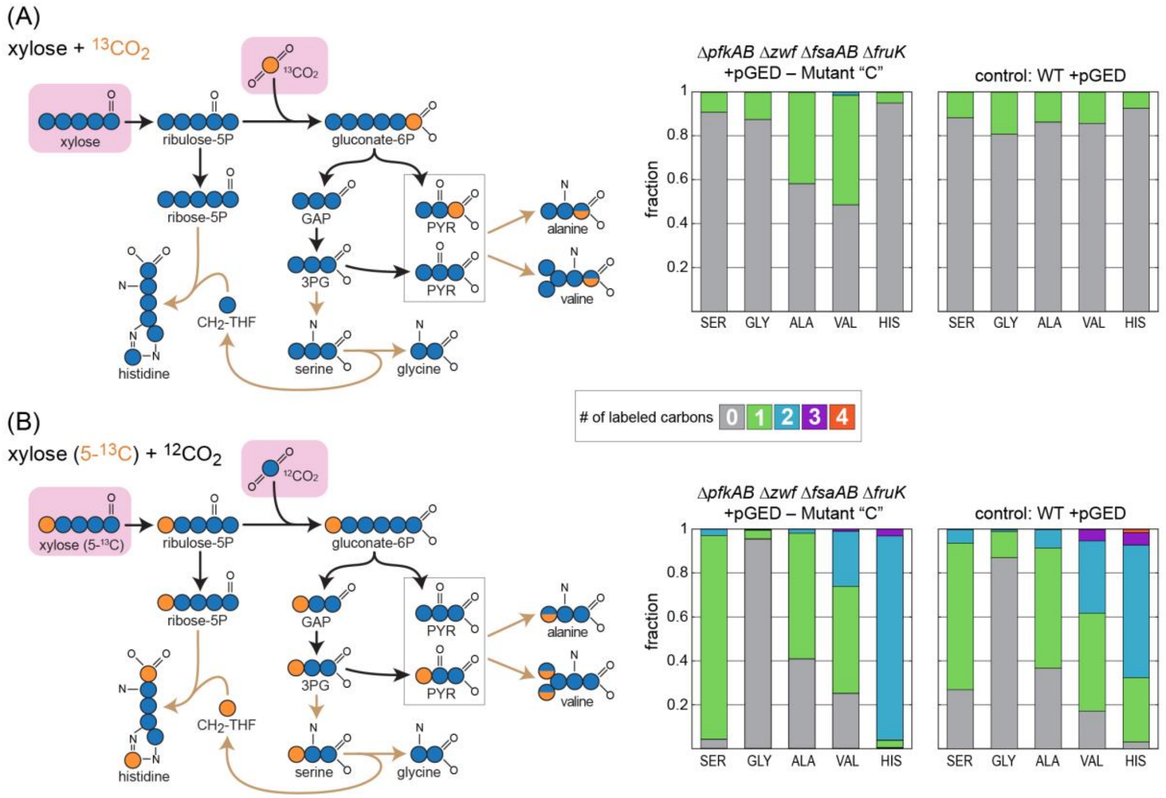

Supplementary Figure 3: ^{13}C-labeling experiments confirm the operation of the GED shunt in a ΔPZF strain ("ΔPZF +pGED Mutant C1"). (A) Cultivation with unlabeled xylose + $^{13}CO2$. (B) Cultivation with xylose (5-^{13}C) + unlabeled CO_2. On the left, the expected labeling distributions for the respective feedstocks are shown. Observed labeling fits the expected pattern and differs from the labeling in a WT strain, which serves as a control. Source data are provided as a Source Data file. Abbreviations: Ala, Alanine; Gly, Glycine; His, Histidine; Ser, Serine; Val, Valine.

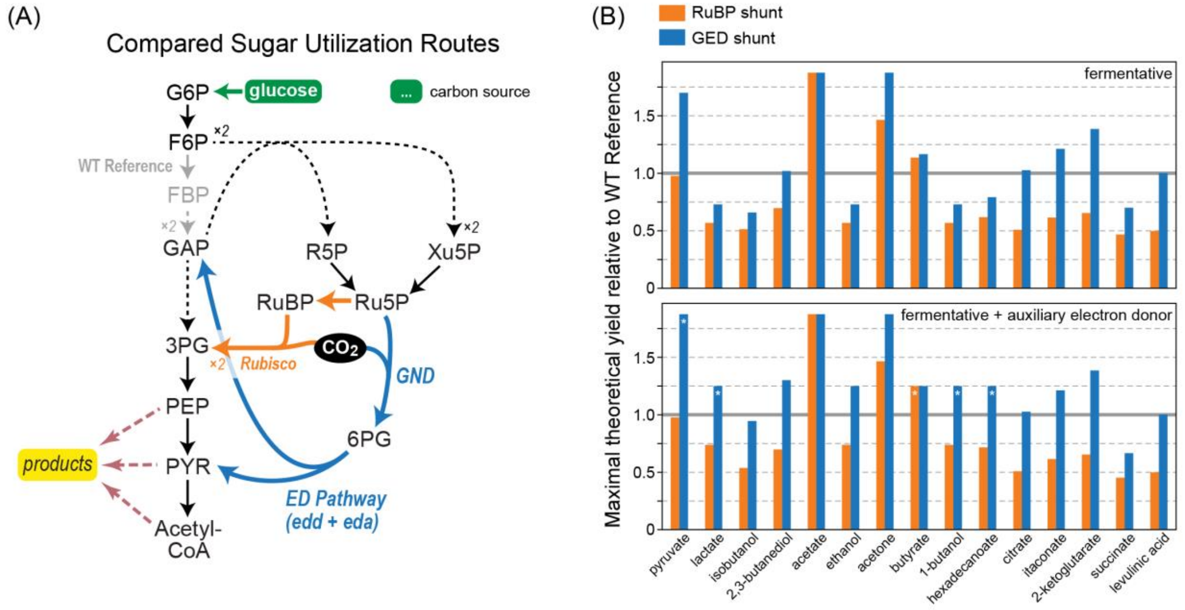

Supplementary Figure 4: Rerouting glucose fermentation via the GED shunt is expected to increase product yields. (A) An overview comparing glucose utilization routes: Canonical utilization (gray) via glycolysis (Embden-Meyerhof-Parnas pathway); the GED shunt (blue); and a linear pathway based on carboxylation by Rubisco ("RuBP shunt", orange). (B) Maximal theoretical yields of 15 fermentation products are shown with or without an additional electron donor, as calculated by Flux Balance Analysis. Presented values are normalized to the product yield from canonical sugar utilization (glycolysis). Most products are predicted to require the secretion of other organic compounds (e.g. acetate, formate) to achieve balancing of reducing equivalents or support ATP biosynthesis. Asterisks denote products that could be produced without byproducts. Source data underlying Supplementary Figure 4B are provided as a Source Data file.

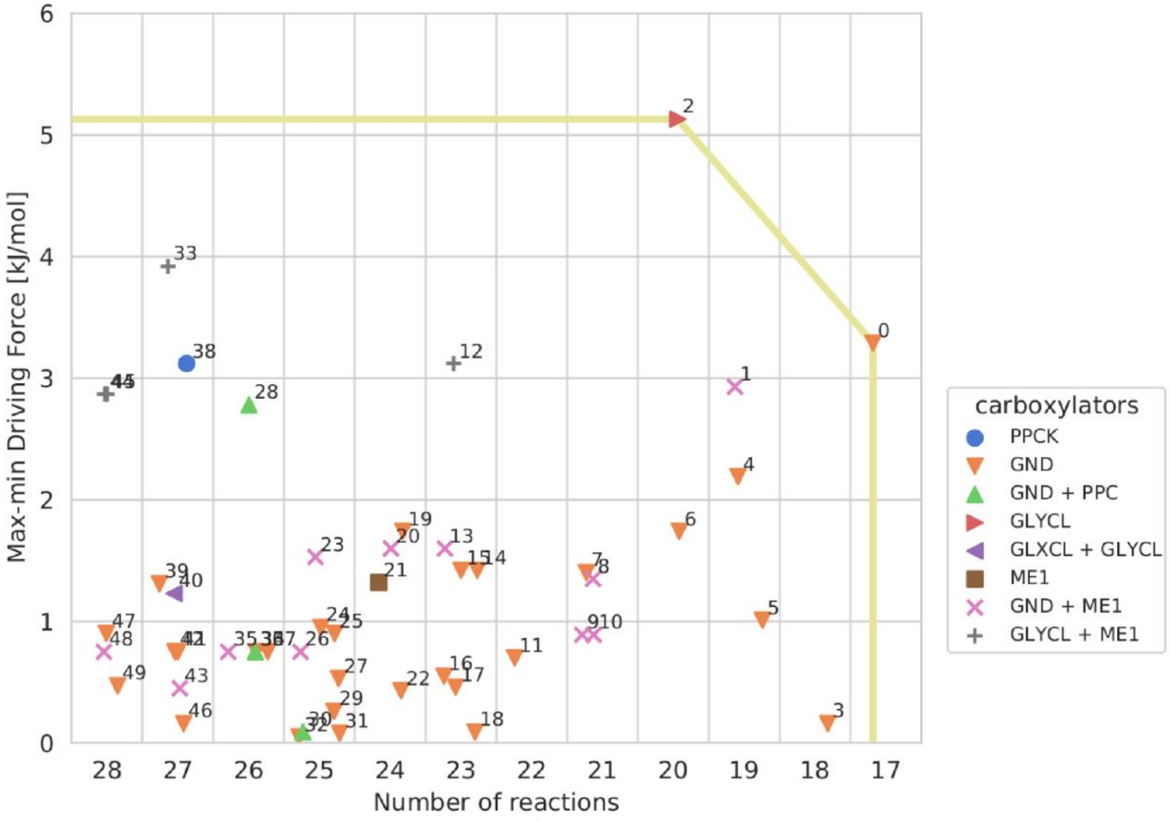

Supplementary Figure 5: The MDF versus the number of reactions for the first 50 pathway solutions. The only two pathways on the Pareto front are 0 and 2. The radius of exclusion around each solution is 3 reactions. The full list of pathways can be found in Supplementary Table S4 and on the GitLab repository. The carboxylating enzymes we found in our pathways are: PPCK (phosphoenolpyruvate carboxykinase), GND (phosphogluconate dehydrogenase), PPC (phosphoenolpyruvate carboxylase), GLYCL (glycine cleavage system), GLXCL (glyoxylate carboxy-lyase), ME1 (malic enzyme, NAD-dependent). The Pareto front is marked by a yellow line.

Supplementary References

[1] Oliver Hädicke, Axel von Kamp, Timur Aydogan, and Steffen Klamt. OptMDFpathway: Identification of metabolic pathways with maximal thermodynamic driving force and its application for analyzing the endogenous CO2 fixation potential of *Escherichia coli*. *PLoS Computational Biology*, **14**, e1006492 (2018).

[2] Jonathan M Monk, Colton J Lloyd, Elizabeth Brunk, Nathan Mih, Anand Sastry, Zachary King, Rikiya Takeuchi, Wataru Nomura, Zhen Zhang, Hirotada Mori, Adam M Feist, and Bernhard O Palsson. iML1515, a knowledgebase that computes *Escherichia coli* traits. *Nature Biotechnology*, **35**, 904–908 (2017).

[3] Elad Noor, Arren Bar-Even, Avi Flamholz, Ed Reznik, Wolfram Liebermeister, and Ron Milo. Pathway thermodynamics highlights kinetic obstacles in central metabolism. *PLoS Computational Biology*, **10**, e1003483 (2014).

[4] Priti Pharkya, Anthony P. Burgard, and Costas D. Maranas. OptStrain: A computational framework for redesign of microbial production systems. *Genome Research*, **14**, 2367–2376 (2004).
