# Supplementary material for: Awakening a latent carbon fixation cycle in Escherichia coli
Source: Nat Commun. 2020 Nov 16;11:5812. doi: 10.1038/s41467-020-19564-5 (PMC7669889; doi:10.1038/s41467-020-19564-5)
Supplement: Supplementary file 3 — Reporting Summary [file 41467_2020_19564_MOESM3_ESM.pdf]

nature research

Corresponding author(s): Steffen Lindner

Last updated by author(s): Oct 7, 2020

Reporting Summary

Nature Research wishes to improve the reproducibility of the work that we publish. This form provides structure for consistency and transparency in reporting. For further information on Nature Research policies, see Authors & Referees and the Editorial Policy Checklist .

Statistics

For all statistical analyses, confirm that the following items are present in the figure legend, table legend, main text, or Methods section.

n/a	Confirmed	
☐	☒	The exact sample size (*n*) for each experimental group/condition, given as a discrete number and unit of measurement
☐	☒	A statement on whether measurements were taken from distinct samples or whether the same sample was measured repeatedly
☒	☐	The statistical test(s) used AND whether they are one- or two-sided<br>*Only common tests should be described solely by name; describe more complex techniques in the Methods section.*
☒	☐	A description of all covariates tested
☒	☐	A description of any assumptions or corrections, such as tests of normality and adjustment for multiple comparisons
☐	☒	A full description of the statistical parameters including central tendency (e.g. means) or other basic estimates (e.g. regression coefficient) AND variation (e.g. standard deviation) or associated estimates of uncertainty (e.g. confidence intervals)
☒	☐	For null hypothesis testing, the test statistic (e.g. *F*, *t*, *r*) with confidence intervals, effect sizes, degrees of freedom and *P* value noted<br>*Give P values as exact values whenever suitable.*
☒	☐	For Bayesian analysis, information on the choice of priors and Markov chain Monte Carlo settings
☒	☐	For hierarchical and complex designs, identification of the appropriate level for tests and full reporting of outcomes
☒	☐	Estimates of effect sizes (e.g. Cohen's *d*, Pearson's *r*), indicating how they were calculated

Our web collection on statistics for biologists contains articles on many of the points above.

Software and code

Policy information about availability of computer code

Data collection	LC-MS data was collected and processed using Thermo Scientific Xcalibur Version 4.1.31.9
Data analysis	The custom algorithm used to identify carbon fixation cycles is extensively described in the methods and Supplementary Information. The source code and all input/output files for reproducing our results are deposited on GitHub (https://gitlab.com/elad.noor/path-designer/tree/master/co2_fixation). Further software used: MatLab R2019a, Python 3.7, COBRApy 0.17, AnnoTree 1.0, equilibrator-api 0.2.5, Geneious 8

For manuscripts utilizing custom algorithms or software that are central to the research but not yet described in published literature, software must be made available to editors/reviewers. We strongly encourage code deposition in a community repository (e.g. GitHub). See the Nature Research guidelines for submitting code & software for further information.

Data

Policy information about availability of data

All manuscripts must include a data availability statement. This statement should provide the following information, where applicable:

- Accession codes, unique identifiers, or web links for publicly available datasets
- A list of figures that have associated raw data
- A description of any restrictions on data availability

Data supporting the findings of this work are available within the paper and its Supplementary Information files. A reporting summary for this Article is available as a Supplementary Information file. The strains reported here are available from the corresponding authors upon reasonable request. The source data underlying all Figures as well as Supplementary Figures is provided as a Source Data file. The complete sequence of the pGED plasmid has been deposited to GenBank (accession MW059023; https://www.ncbi.nlm.nih.gov/genbank/). Where indicated, data from the following public repositories was used for the findings in this study: KEGG (https://www.kegg.jp/); BiGG (http://bigg.ucsd.edu/); eQuilibrator (http://equilibrator.weizmann.ac.il/).

Field-specific reporting

Please select the one below that is the best fit for your research. If you are not sure, read the appropriate sections before making your selection.

[x] Life sciences [ ] Behavioural & social sciences [ ] Ecological, evolutionary & environmental sciences

For a reference copy of the document with all sections, see nature.com/documents/nr-reporting-summary-flat.pdf

Life sciences study design

All studies must disclose on these points even when the disclosure is negative.

Sample size	Unless specified otherwise, most experiments were performed in triplicate, a commonly chosen sample size to ensure biological reproductibility. For growth curves, shown results are single experiments representative of at least three repititions showing similar results. Technical duplicates, triplicates or quadruplicates were used for growth curves, as denoted in the text.
Data exclusions	No data were excluded from the analysis.
Replication	Reproducibility was verified by conducting growth experiments at least three times in culture tubes and microplate readers, with freshly prepared cells from glycerol stocks.
Randomization	For biological replicates, independent single clones were chosen at random from plates.
Blinding	Blinding was performed in the analysis of data from the isotopic labeling experiments and qRT-PCR. Blinding was not performed for growth experiments.

Reporting for specific materials, systems and methods

We require information from authors about some types of materials, experimental systems and methods used in many studies. Here, indicate whether each material, system or method listed is relevant to your study. If you are not sure if a list item applies to your research, read the appropriate section before selecting a response.

Materials & experimental systems

n/a	Involved in the study
[x]	[ ] Antibodies
[x]	[ ] Eukaryotic cell lines
[x]	[ ] Palaeontology
[x]	[ ] Animals and other organisms
[x]	[ ] Human research participants
[x]	[ ] Clinical data

Methods

n/a	Involved in the study
[x]	[ ] ChIP-seq
[x]	[ ] Flow cytometry
[x]	[ ] MRI-based neuroimaging
