# Supplementary material for: Awakening a latent carbon fixation cycle in Escherichia coli
Source: Nat Commun. 2020 Nov 16;11:5812. doi: 10.1038/s41467-020-19564-5 (PMC7669889; doi:10.1038/s41467-020-19564-5)
Supplement: Supplementary file 4 — Description of Additional Supplementary Files [file 41467_2020_19564_MOESM4_ESM.pdf]

Description of Additional Supplementary Files

Supplementary Data 1

Sequence of the genomic region upstream of the *pntA* gene in the isolated *ΔtktAB* + pGED mutant strain. Binding sites of the primers used for Sanger sequencing are in bold; coding sequence of the pntA gene is underlined; sequence of the IS5 mobile element is shown in red.

Supplementary Data 2

Sequence of the construct (linear dsDNA) used for *pntA* promotor engineering with the λ Red recombinase method. Homologous regions for recombination are in bold lower-case; introduced promotor region is in upper-case; ribosome binding site + *pntA* start codon are underlined; CmR selection marker is shown in red.
