# Supplementary material for: Awakening a latent carbon fixation cycle in Escherichia coli
Source: Nat Commun. 2020 Nov 16;11:5812. doi: 10.1038/s41467-020-19564-5 (PMC7669889; doi:10.1038/s41467-020-19564-5)
Supplement: Supplementary file 2 — Peer Review File [file 41467_2020_19564_MOESM2_ESM.pdf]

Reviewers' comments:

Reviewer #1 (Remarks to the Author):

The manuscript by Satanowski et al. describes the identification and efforts to implement native E. coli enzymes comprising a potential carbon fixation pathway. The authors use a computational strategy based on the genome scale model of E. coli to identify potential pathways resulting in the conversion of CO2 to pyruvate. Key to this approach was the assumption that all reactions are reversible, with thermodynamic driving force applied instead as the main determinant of feasibility. This led to the identification of a pathway based on the reversal of the reaction catalyzed by 6-phosphogluconate dehydrogenase (Gnd), an enzyme of the oxidative branch of the pentose phosphate pathway. The authors demonstrate that Gnd is reversible in vitro and in vivo, by enabling growth with a pentose co-substrate in a strain that is deficient in the ability to consume pentose sugars.

The work by Satanowski et al. is interesting for its insight into the computational approach to identify 'latent' pathways in metabolism that are significantly underexplored. As the authors suggest, these types of pathways may operate in nature, although this claim is so far unsupported. While certainly significant in this sense, the experimental approach left some to be desired especially in regards to: 1) novelty, as Gnd has been demonstrated to be reversible by others (see for example Biochemistry 32, 2036-2040, 1993; Methods Enzymol 41, 214-220, 1975), something that also happens in similar enzymes like malic enzyme and isocitrate dehydrogenase; and 2) in light of other recent work that used similar approaches to implement pentose phosphate rearrangement pathways (e.g. RuMP) in E. coli. In the same sense, the Δrpe and ΔtktAB approaches were highly similar conceptually, and although one approach led to additional insight into the levers controlling the pathway function, it would have been nice to see something different.

Other specific comments:
- The authors should provide some rationale for the selection of pyruvate as the arbitrary product used in the computational analysis? Would the authors expect a different result from the selection of a different product (especially for example, a C2 molecule such as acetyl-CoA)? Wouldn't biomass be a more generalizable product selection within the context of the genome scale model and with the objective of growth in mind?
- The authors should explain what they mean in the case of "no growth"? The growth curves in Figure 3 lead to some confusion on this. While I agree that there is clearly very little growth for example in the brown and purple curves in panel D (especially relative to the strains that do grow), there seems to be something there relative to the strains grown in ambient CO2 conditions, which are very clearly no growth. Perhaps there is increased evaporation due to the difference in headspace composition (although Figure 2B seems to indicate this is not the case)? Control experiments in the 20% CO2 headspace with no additional C5 carbon source may be useful to clarify this phenomenon.

Reviewer #2 (Remarks to the Author):

This paper examines the presence and activation of latent CO2-fixing pathways in E coli using only endogenous enzymes. A key aspect of the paper is that the authors infer rather than assume directionality of all reactions by allowing metabolite concentrations (and thus the thermodynamic driving forces) of reactions to vary. They use this approach to identify potential CO2-fixing pathways that have previously been missed. This perspective is a big part of what makes this paper exciting. The importance of considering how varying metabolite concentrations affects the driving forces and directionality of reactions in the context of the networks they make up has been increasingly recognized in recent years, and is revising our understanding on the origin, evolution and (as the authors nicely here show) engineering of metabolism.

Computational predictions were followed up through a series of experiments in which the authors show the feasibility of the pathway they identified. Specifically, they showed that genetic manipulations that either increase the concentration of inputs, or decrease the concentration of outputs, of the central CO2-fixing reaction they identified enabled it to be driven forward. While the authors do not show operation of full autotrophic growth because of the myriad other subtle changes that will likely be needed for such a wholesale change to the global state of metabolism, the ability to drive the central CO2-fixing reaction forward shows its feasibility. The authors further performed an evolutionary experiment in which one of the key modifications (increasing the supply of NADPH) needed to drive CO2-fixation forward was achieved through a genetic change. This highlights how adaptive laboratory evolution provides a general path forward for achieving full autotrophic growth using the pathway they identify.

The paper is well written, the questions being addressed are timely and interesting, the analyses and experiments are thorough and the conclusion justified, and so I am supportive of it. I think only minor revisions should be needed. See comments below.

Rogier Braakman

Comments:

Abstract - There a few small things in the abstract that could perhaps be a bit clearer. The authors may want to state earlier on in the paper what Gnd is - as it stands the name of the GED cycle is an acronym of an acronym and it isn't easy to visualize the pathway from what is written. Perhaps the sentence "This autocatalytic route is based upon…" could be expanded just a bit so that readers can more easily understand the ways in which this pathway is a patchwork of existing things (i.e. reversal of a key oxidative pentose phosphate pathway reaction coupled to Entner-Doudoroff variant of glycolysis {etc}, or some such description).

Abstract - Similarly, the authors may want to expand just a bit more on the linear GED shunt and how it differs from the cycle, so that the reader can more clearly understand what has and hasn't been achieved, e.g. "- a linear variant of the pathway in which the CO2-fixing is active but its input substrate cannot be regenerated".

Lines 32-34: why is achieving synthetic autotrophy an important goal? It might help readers to provide some more context. More generally, what was your motivation for this work? Is it related to sustainability efforts, agricultural efforts, basic understanding of metabolic evolution or something else? This becomes clearer later in the paper, but it helps frame why you are looking for the pathway features you seem to be looking for and how you design your analyses

Line 34-38: do the authors want to add anything about pros and cons of different pathways? I could see how for example rate/yield tradeoffs might be important to consider, but in different ways depending on your goals. Otherwise you run the risk that readers could wonder why you are looking at yet another pathway for doing something similar to what others have already achieved in other ways

Lines 47-49: the ending of this paragraph suggests that understanding evolution is a major motivation, which I'm not sure it was?

Lines 57-58: as in the abstract the distinction between the GED cycle and shunt is unclear and is confusing me. Perhaps you want to include a figure showing both already in the introduction, or else maybe you want to clarify and explain a bit more

Line 73: I think the natural habitat of E coli is much broader than just the human gut, they are found in other environments too, no? Or was the particular strain you focused on isolated from the gut?

Line 78: Why did you prefer fewer enzymes in your optimization criterium? Also, the text in the method section suggests that changing the weight between your criteria did not change your main results.

Line 79: why did you choose a cutoff of >3 kJ/mole? I guess the more general point here is that you might want to explain to your reader why you designed the optimization process the way you did – what was the underlying motivation or question? I could see how you might choose different criteria depending on your goal

Lines 96-97: why does oxygen sensitivity and complexity make other variants less attractive? Also, in what sense are they complex (seems a bit abstract)? This goes toward understanding your motivation

Line 120: It might be interesting for the reader to have this number of 200 mbar placed in context – how does it compare to what you might see in a hydrothermal vent or in earlier eras of Earth history when [CO2] is thought to have been a lot higher? This might not be the place for this discussion, but I wanted to flag it here since it came to mind already at this point… I late noticed you have some discussion on this kind of point in the discussion

Line 130: I first read this as 3% of current atmospheric CO2 levels, i.e. this is essentially always achieved. I soon realized this was wrong, but perhaps citing value of what the current atmosphere contains could help as general context

Line 144-148: I'm again getting confused about some of the details of the GED shunt and its relation to the cycle… you explain how the shunt has all the reactions of the cycle except that it lacks the ability to regenerate the substrate Ru5P due to the deletion of RPE. But there is also still R5P isomerase, which of course does produce Ru5P so it's not immediately clear to me how the RPE mutant only possesses a shunt and not a cycle… Said another way it's not clear to me why RPE is critical for the operation of the GED cycle in general, and so similarly it's not clear to me that you could not in theory get the RPE mutant to grow on only CO2 if other subtle adaptation (like those you refer to elsewhere) fell into place. To be clear, I do follow how your experiments show the ability of driving Gnd to fix CO2, that is in fact quite clear and elegant, my confusing is coming in around the cycle vs shunt discussion and why you are calling something a shunt.

Figure 2: in the context of the previous point please carefully double check the direction of all your arrows and the names of your metabolites. Due to my confusion around the cycle vs shunt discussion I was for a while wondering whether you had swapped the R5P and Ru5P labels… Also, several arrows have different directions in Fig. 2 and 3, and shouldn't many of those reactions be bidirectional anyway? (you are showing results from different reactions that push the network in different directions in both cases).

Line 163: Why do you say no growth? The right panel shows a positive slope at the beginning of the experiment even under ambient CO2. Not much perhaps, but there does appear to be some growth. This is a point that comes back in many if not most of your figures…

Line 167-168: I don't see any blue lines in Fig. 2B. I do see brown lines and upon closer inspection those appear to be the experiment in question. Again there is the point of a positive slope when you mention no growth…

Lines 175-177: you might want to discuss your WT in the text. Partly because your observations are made against this control and so seems like they should be discussed, but I am also wondering about the observed patterns (always some labeling of one carbon, and only one carbon, in all the compounds). Do you think this is because of anaplerotic reactions in the TCA cycle causing the label to end up everywhere? This is a minor point.

Line 181: case in point about the cycle vs shunt confusion, here you delete another gene than RPE, so it is again not clear to what exactly the shunt is. It seems like perhaps you mean a group of variant pathways rather than a single thing, but it is still a bit confusing. At least in this case it is more obvious how the TKT mutant would not be able to fix CO2 without supplementation of other organics .

[end note: after completing the review and thinking it over i realized that with GED shunt you mean simply the core sequence of Gnd leading into ED glycolysis, and the cycle contains the shunt but then regenerates multiples of its inputs from the outputs. It would be good to make this really clear throughout. Similarly the confusion around whether it is correct to describe the RPE mutant as not having a complete (latent) cycle should be given some thought, as outlined above]

Line 184: it is not just the reductive oxidative pentose phosphate pathway but both the reductive and oxidative variants that are abolished in the mutant, no? Also probably better to say 'reductive' rather than 'non-oxidative'…

Line 263-264: I again feel like the authors might want to say something about their WT control, even if just to acknowledge that the pattern is much more complex than compared to the WT that is given labeled CO2. It's just a very noticeable and elaborate pattern and so readers might appreciate some basic intuitive guide to make sense of it. But this is again a minor point.

Line 281 and surrounding text: this CETCH acronym does not tell us much about the nature of the cycle. Perhaps the authors could say a bit more, like some of the different partial pathways it makes use of, to highlight why it should be considered 'quite complex', which without context feels a bit subjective

Lines 298-299: Maybe remind the reader what this is rather than relying on acronyms that require the reader to look for it. E.g. "support growth of mutant that lacked the ability to convert X to Y, but not a mutant that lacked the ability to convert Z to Y, even though both disrupt the pentose phosphate pathway"

Reviewer #3 (Remarks to the Author):

In this work, the authors have demonstrated a new CO2 fixation route in E. coli by overexpressing three endogenous genes (gnd, edd, eda) and deleting one (rpe) or two (tktA and tktB) other genes. The new pathway was termed the "GED shunt". The net stoichiometry of the GED shunt is the condensation of one CO2 molecule and one pentose sugar (either ribose or xylose) to produce pyruvate and a three carbon sugar-phosphate. The operation of the GED shunt was conclusively verified using 13C tracer experiments. The authors suggest that the GED shunt is the first step towards the establishment of a "GED cycle" that could fix much more CO2.

Major concerns:

1) The GED shunt has little or no practical use, since it relies on expensive substrates (e.g. ribose) to fix a small amount of CO2, i.e. at most 17% of carbon used for cell growth or product formation is derived from fixed CO2. Other researchers have already engineered E. coli to efficiently fix CO2 using the Calvin cycle, where 100% of carbohydrates in biomass are derived from fixed CO2.

2) Throughout the manuscript, the authors suggest that the GED shunt can be eventually evolved into the cyclic GED cycle, to allow more CO2 fixation. However, there is absolutely no experimental evidence presented in this manuscript that demonstrates that this can be achieved with the current strains. In fact, careful inspection of the reaction required for the GED cycle suggests that the

described strains used for GED shunt can never be used for the GED cycle, since the GED-shunt strains are missing key enzymes (i.e. rpe and tktAB) that are required for the operation of the GED cycle. Thus, contrary to the suggestion of the authors, the strains reported in this work cannot be evolved to establish the GED cycle. This greatly diminishes the significance of the present study. It is essentially a dead-end from a pathway engineering perspective.

Awakening a latent carbon fixation cycle in *Escherichia coli*

Answers to reviewer's comments

**Reviewer #1 (Remarks to the Author)**:

The manuscript by Satanowski et al. describes the identification and efforts to implement native E. coli enzymes comprising a potential carbon fixation pathway. The authors use a computational strategy based on the genome scale model of E. coli to identify potential pathways resulting in the conversion of CO2 to pyruvate. Key to this approach was the assumption that all reactions are reversible, with thermodynamic driving force applied instead as the main determinant of feasibility. This led to the identification of a pathway based on the reversal of the reaction catalyzed by 6-phosphogluconate dehydrogenase (Gnd), an enzyme of the oxidative branch of the pentose phosphate pathway. The authors demonstrate that Gnd is reversible in vitro and in vivo, by enabling growth with a pentose co-substrate in a strain that is deficient in the ability to consume pentose sugars.

The work by Satanowski et al. is interesting for its insight into the computational approach to identify 'latent' pathways in metabolism that are significantly underexplored. As the authors suggest, these types of pathways may operate in nature, although this claim is so far unsupported.

We thank the reviewer for the support.

While certainly significant in this sense, the experimental approach left some to be desired especially in regards to: 1) novelty, as Gnd has been demonstrated to be reversible by others (see for example Biochemistry 32, 2036-2040, 1993; Methods Enzymol 41, 214-220, 1975), something that also happens in similar enzymes like malic enzyme and isocitrate dehydrogenase;

We appreciate that the reductive carboxylation activity of Gnd was measured before *in vitro*. We indeed give credit to these previous studies in the text. However, we argue that there is a vast difference between measuring a reaction using a purified enzyme versus confirming its relevance *in vivo*. In fact, many reactions can be detected *in vitro* that could never work *in vivo* due to additional constraints on the concentrations of substrates and products. For example, the kinetic characterization of Gnd by previous studies and in our own work was performed in absence of the reaction products ($NADP^+$ and 6P-gluconate) to measure initial reaction rates. In the cell, however, a reaction must operate at the presence of both substrate and products, and the relative concentration of cofactors, that is, [NADP]/[$NADP^+$], is rather fixed and cannot be easily modified to support the required directionality.

Hence, from our perspective, **the novelty of our work stems from the fact that we demonstrated that reductive carboxylation by Gnd is not only able to proceed under physiological constraints, but also, that it can sustain almost all cellular carbon flux**. Importantly, this reductive activity has relevance in the context of potential carbon fixation pathways. We would like to point the reviewer's attention to the recent description of a variant of the reductive TCA cycle, which shows similarities to the findings in our work (Mall *et al.* Science 2017; Nunoura *et al.* Science 2017). Although it was long known that the reaction of citrate synthase in the TCA cycle is reversible *in vitro*, it remained long doubted that this reaction could also operate in the reverse *in vivo* and moreover sustain sufficient flux for synthesis of all biomass.

Furthermore, the reviewer is correct that malic enzymes and isocitrate dehydrogenase have indeed been shown to operate in the reductive direction both *in vitro* and *in vivo* (which we also refer to in the text). In fact, we would argue that given the analogy to these enzymes, it is surprising how commonly Gnd is still portrayed as an irreversible decarboxylase.

Finally, we note that the kinetic parameters of *E. coli*'s Gnd, measured in this study, are substantially better than those previously measured for a eukaryotic enzyme.

Following the reviewer's comments, we modified the following sentences in the text:

"While sporadic studies have reported that some Gnd variants support the reductive carboxylation of Ru5P *in vitro* [30-34], a comprehensive kinetic characterization of this activity in bacterial Gnd variants is lacking. More importantly, it remains unclear whether this reaction could operate under physiological conditions, where the concentrations of substrates and products are constrained; that is, substrate concentrations are not necessarily saturating and product concentrations are non-negligible."

"Notably, these kinetic parameters are substantially better than those previously reported for a eukaryotic Gnd variant ($k_{cat}$ ~ 1 $s^{-1}$ and $K_M(CO_2) \geq 15$ mM [30-31])"

30. Villet, R.H. & Dalziel, K. The nature of the carbon dioxide substrate and equilibrium constant of the 6-phosphogluconate dehydrogenase reaction. Biochem J 115, 633-638 (1969).
31. Villet, R.H. & Dalziel, K. Studies of 6-phosphogluconate dehydrogenase from sheep liver. 1. Kinetics of the reductive carboxylation reaction. Eur J Biochem 27, 244-250 (1972).
32. Silverberg, M. & Dalziel, K. 6-Phospho-D-gluconate dehydrogenase from sheep liver. Methods Enzymol 41, 214-220 (1975).
33. Berdis, A.J. & Cook, P.F. Overall kinetic mechanism of 6-phosphogluconate dehydrogenase from Candida utilis. Biochemistry 32, 2036-2040 (1993).
34. Hanau, S., Montin, K., Cervellati, C., Magnani, M. & Dallocchio, F. 6-Phosphogluconate dehydrogenase mechanism: evidence for allosteric modulation by substrate. J Biol Chem 285, 21366-21371 (2010).

and 2) in light of other recent work that used similar approaches to implement pentose phosphate rearrangement pathways (e.g. RuMP) in E. coli. In the same sense, the ∆rpe and ∆tktAB approaches were highly similar conceptually, and although one approach led to additional insight into the levers controlling the pathway function, it would have been nice to see something different.

The reviewer is correct that in the work of other groups as well as in one of our recent studies, the ∆rpe and ∆tktAB strains were used to demonstrate the activity of the RuMP shunt in *E. coli* (we indeed cite these studies in the text). These strains by themselves do not represent a novelty. However, we view them only as a tool, where the novelty lies in the carbon fixation route we designed/discovered and tested. In fact, we feel that the use of the ∆rpe and ∆tktAB strains, which were already characterized in detail, adds credibility to our approach, claims, and findings.

Still, following also a comment from another reviewer, we have now added a third selection scheme, which would enable a smooth transition from the GED shunt to the GED cycle (and was not used to establish the RuMP shunt).

Other specific comments:

- The authors should provide some rationale for the selection of pyruvate as the arbitrary product used in the computational analysis? Would the authors expect a different result from the selection of a different product (especially for example, a C2 molecule such as acetyl-CoA)? Wouldn't biomass be a more generalizable product selection within the context of the genome scale model and with the objective of growth in mind?

We agree with the reviewer that the choice of pyruvate as a product seems arbitrary. However, in fact, **the choice of the specific product does not add or remove candidate pathways from the list, as all metabolites in central metabolism are connected and can be converted into each other** (e.g., all cycles identified to generate pyruvate would also be identified to generate acetyl-CoA and vice versa as acetyl-CoA can be converted to pyruvate via the glyoxylate shunt). While it is still true that the ranking of the pathways (by number of reactions and driving force) might somewhat change by defining a different product, **we chose pyruvate since it is commonly used as a reference point by which carbon fixation pathways are compared** (following the reviewer's comment, we now added a reference to this point in the text.)

Furthermore, conversion of pyruvate to other metabolic intermediates requires much fewer steps than conversion of e.g. acetyl-CoA which relies on the glyoxylate shunt under aerobic conditions; therefore, choosing acetyl-CoA as product would unfairly improve the ranking of pathways that directly produce this compound, ignoring the fact that its further assimilation is rather costly with regard to the number of required reactions.

We also wish to clarify that using biomass as product would not work in our case: (i) it would be technically difficult to identify which exact reactions participate in the carbon fixation cycle, that is, which reactions are part of it and which just lead to biomass building blocks; and (ii) it would be impossible to rank the pathways the way we did as the number of reactions will be ill-defined for such a huge network and the MDF might be constrained by biosynthetic routes rather than the carbon fixation pathway itself.

- The authors should explain what they mean in the case of "no growth"? The growth curves in Figure 3 lead to some confusion on this. While I agree that there is clearly very little growth for example in the brown and purple curves in panel D (especially relative to the strains that do grow), there seems to be something there relative to the strains grown in ambient CO2 conditions, which are very clearly no growth. Perhaps there is increased evaporation due to the difference in headspace composition (although Figure 2B seems to indicate this is not the case)? Control experiments in the 20% CO2 headspace with no additional C5 carbon source may be useful to clarify this phenomenon.

The reviewer has raised a good point. Indeed, some of the strains we claim not to grow do show a slight increase in OD. (We note that our use of a log-scale makes such minute growth seem quite substantial although the actual increase in OD is very small). We are certain that this OD increase is not due to evaporation as we always use mineral oil, which, from our vast experience in these experiments, makes evaporation completely negligible even after a week or more of cultivation (while oxygen and $CO_2$ can easily penetrate it).

We emphasize that those strains that show the 'minute growth' were all constrained to less than two doublings (starting from an OD of 0.02 and ending at an OD lower than 0.08). While we cannot provide a concrete answer for why these 1-2 doublings did occur, we can speculate that it might be related to inefficient use of the GED shunt that, while operating, could not support continued growth. Indeed, in all instances where we observe a 'minute growth' phenotype, it could be potentially explained by a use of the GED shunt (e.g., all were at high $CO_2$ concentration and expressing the GED shunt enzymes).

Following the reviewer's comment, we have amended the text in all relevant instances to clarify that by 'no growth' we mean less than 2 doublings.

**Reviewer #2 (Remarks to the Author)**:

This paper examines the presence and activation of latent CO2-fixing pathways in E coli using only endogenous enzymes. A key aspect of the paper is that the authors infer rather than assume directionality of all reactions by allowing metabolite concentrations (and thus the thermodynamic driving forces) of reactions to vary. They use this approach to identify potential CO2-fixing pathways that have previously been missed. This perspective is a big part of what makes this paper exciting. The importance of considering how varying metabolite concentrations affects the driving forces and directionality of reactions in the context of the networks they make up has been increasingly recognized in recent years, and is revising our understanding on the origin, evolution and (as the authors nicely here show) engineering of metabolism.

Computational predictions were followed up through a series of experiments in which the authors show the feasibility of the pathway they identified. Specifically, they showed that genetic manipulations that either increase the concentration of inputs, or decrease the concentration of outputs, of the central CO2-fixing reaction they identified enabled it to be driven forward. While the authors do not show operation of full autotrophic growth because of the myriad other subtle changes that will likely be needed for such a wholesale change to the global state of metabolism, the ability to drive the central CO2-fixing reaction forward shows its feasibility. The authors further performed an evolutionary experiment in which one of the key modifications (increasing the supply of NADPH) needed to drive CO2-fixation forward was achieved through a genetic change. This highlights how adaptive laboratory evolution provides a general path forward for achieving full autotrophic growth using the pathway they identify.

The paper is well written, the questions being addressed are timely and interesting, the analyses and experiments are thorough and the conclusion justified, and so I am supportive of it. I think only minor revisions should be needed. See comments below.

Rogier Braakman

We thank the reviewer for his support and nice words. We are happy that he appreciates our approach, experiments, and findings. We also appreciate all the constructive comments which helped us improve the manuscript.

Comments:

Abstract - There a few small things in the abstract that could perhaps be a bit clearer. The authors may want to state earlier on in the paper what Gnd is - as it stands the name of the GED cycle is an acronym of an acronym and it isn't easy to visualize the pathway from what is written. Perhaps the sentence "This autocatalytic route is based upon…" could be expanded just a bit so that readers can more easily understand the ways in which this pathway is a patchwork of existing things (i.e. reversal of a key oxidative pentose phosphate pathway reaction coupled to Entner-Doudoroff variant of glycolysis {etc}, or some such description).

We agree with the reviewer that a clarification of the sentence would be useful. As suggested, we changed the sentence to say: "This autocatalytic route is based on reductive carboxylation of ribulose 5-phosphate (Ru5P) by 6-phosphogluconate dehydrogenase (Gnd), followed by reactions of the Entner-Doudoroff pathway, gluconeogenesis, and the pentose phosphate pathway"

Abstract - Similarly, the authors may want to expand just a bit more on the linear GED shunt and how it differs from the cycle, so that the reader can more clearly understand what has and hasn't been achieved, e.g. "- a linear variant of the pathway in which the CO2-fixing is active but its input substrate cannot be regenerated".

We agree with the reviewer and changed the sentence accordingly: "We demonstrate the *in vivo* feasibility of this new-to-nature pathway by constructing *E. coli* gene deletion strains whose growth on pentose sugars depends on the GED shunt, a linear variant of the GED cycle which does not require the regeneration of Ru5P". Due to limitations on the length of the abstract we could not explain in detail the logic behind the shunt. Instead, we explain it in detail in the text (see below).

Lines 32-34: why is achieving synthetic autotrophy an important goal? It might help readers to provide some more context. More generally, what was your motivation for this work? Is it related to sustainability efforts, agricultural efforts, basic understanding of metabolic evolution or something else? This becomes clearer later in the paper, but it helps frame why you are looking for the pathway features you seem to be looking for and how you design your analyses.

Following the reviewer's comments we now added a statement regarding the biotechnological application of synthetic autotrophy: "...with the long-term goal of achieving synthetic autotrophy, which could pave the way towards sustainable bioproduction schemes rooted in $CO_2$ and renewable energy"

In fact, our motivation was more focused on the fundamental/evolutionary question of whether it is possible to harness the endogenous enzymes of an obligate heterotroph to achieve autotrophy. We indeed say: "The limited number of natural carbon fixation pathways might indicate that the recruitment of endogenous enzymes to support carbon fixation is a rather exceptional event. To understand this process better we aimed to recreate it in a modern heterotrophic bacterium."

Line 34-38: do the authors want to add anything about pros and cons of different pathways? I could see how for example rate/yield tradeoffs might be important to consider, but in different ways depending on your goals. Otherwise you run the risk that readers could wonder why you are looking at yet another pathway for doing something similar to what others have already achieved in other ways

We believe that the pros and cons of different pathways are less relevant for this study. We are not primarily interested in identifying a superior pathway, but rather in exploring the feasibility of recreating the emergence of a novel carbon fixation pathway from the recruitment of endogenous enzymes of a heterotroph.

Lines 47-49: the ending of this paragraph suggests that understanding evolution is a major motivation, which I'm not sure it was?

In fact, it is exactly the primary interest of this paper: "The limited number of natural carbon fixation pathways might indicate that the recruitment of endogenous enzymes to support carbon fixation is a rather exceptional event. To

understand this process better we aimed to recreate it in a modern heterotrophic bacterium." / "Our findings indicate the feasibility of recruiting endogenous enzymes to establish a novel carbon fixation pathway and pave the way for future establishment of synthetic autotrophy based on new-to-nature pathways."

Lines 57-58: as in the abstract the distinction between the GED cycle and shunt is unclear and is confusing me. Perhaps you want to include a figure showing both already in the introduction, or else maybe you want to clarify and explain a bit more

Following the reviewer's comment, we refer to figure 2A when discussing the shunt in more detail. We have amended the sentence here to better explain the meaning of the shunt: "We demonstrate that overexpression of key pathway enzymes together with small modifications of the endogenous metabolic network enable growth via the "GED shunt" – a linear route that requires the key reactions of the GED cycle, including the carboxylation step, for the biosynthesis of (almost) all biomass building blocks"

Line 73: I think the natural habitat of E coli is much broader than just the human gut, they are found in other environments too, no? Or was the particular strain you focused on isolated from the gut?

The main habitats of *E. coli* are indeed the lower intestines of warm-blooded animals. To avoid strong phrasing, we replaced "i.e." with "e.g." and "human" with "mammalian": "the natural habitats of *E. coli*, e.g., the mammalian gut"

Line 78: Why did you prefer fewer enzymes in your optimization criterium? Also, the text in the method section suggests that changing the weight between your criteria did not change your main results.

Pathways with fewer enzymes are easier to engineer, to troubleshoot, and to analyze. Shorter pathways are also less likely to suffer from deleterious clashes with flux in endogenous metabolism.

We now shortly relate to these issues in the text: "We ranked the pathways according to two key criteria that can be calculated for each of them in a straightforward manner: their MDF and the number of enzymes they require (preferring fewer enzymes, see Methods and Supplementary Text). Pathways ranked high in terms of these criteria are expected to be simpler to establish and to operate more robustly under fluctuating physiological conditions."

Line 79: why did you choose a cutoff of >3 kJ/mole?

We did not use 3 kJ/mol as a cutoff. Rather, we just report the MDF of the GED cycle. We actually present all pathways with positive MDF (as we show in the Supplementary Information).

We further add that at in a previous study (Noor, E., Bar-Even, A., Flamholz, A., Reznik, E., Liebermeister, W. and Milo, R., 2014. Pathway thermodynamics highlights kinetic obstacles in central metabolism. PLoS Comput Biol, 10(2), p.e1003483.) we showed that having a driving force >3 kJ/mol ensures that the Force Flux efficacy, that is, the ratio between the net reaction flux ($J^+$-$J^-$) and the total flux ($J^+$+$J^-$), is >50%. We now added this information to the text: "...> 3 kJ/mol, such that reverse enzyme flux is minimal [13]"

I guess the more general point here is that you might want to explain to your reader why you designed the optimization process the way you did – what was the underlying motivation or question? I could see how you might choose different criteria depending on your goal

The reviewer is indeed correct that multiple criteria can be used to compare and analyze the pathways. However, some of these – for example, kinetics and integration with endogenous metabolism – cannot be easily calculated for all candidate pathways, making the analysis very difficult. Other criteria might be easier to calculate, for example, ATP-efficiency. However, we felt that this criterion is actually of less importance in the current analysis as we do not try to find an optimal pathway, but rather the simplest and most robust one that can be reconstructed from the endogenous enzymes of the host.

Following the reviewer's comment, we now shortly refer to this point in the text: "We ranked the pathways according to two key criteria that can be calculated for each of them in a straightforward manner: their MDF and the number of enzymes they require (preferring fewer enzymes, see Methods and Supplementary Text). Pathways ranked high in

terms of these criteria are expected to be simpler to establish and to operate more robustly under fluctuating physiological conditions."

Lines 96-97: why does oxygen sensitivity and complexity make other variants less attractive? Also, in what sense are they complex (seems a bit abstract)? This goes toward understanding your motivation

Restricting autotrophic growth to anaerobic conditions would severely limit the applicability and flexibility of the pathway and would further considerably complicate our technical work. The other pathways are more complex in terms of their number of enzymes but also in terms of general structure (having multiple embedded cycles) and overlap with central metabolism. These pathways would therefore be extremely difficult to establish. However, we prefer not to elaborate on these issues in the text as we feel it would make it harder for the general reader. Instead, we send the reader to view the structure of these alternative cycles in Fig. 1B,C and judge for themselves.

Line 120: It might be interesting for the reader to have this number of 200 mbar placed in context – how does it compare to what you might see in a hydrothermal vent or in earlier eras of Earth history when [CO2] is thought to have been a lot higher? This might not be the place for this discussion, but I wanted to flag it here since it came to mind already at this point… I late noticed you have some discussion on this kind of point in the discussion

As we wrote earlier in the text: "We assumed an elevated $CO_2$ concentration of 20% (200 mbar), which is easily attainable in microbial cultivation within an industrial context and further characterizes the natural habitats of *E. coli*, e.g., the mammalian gut [15,16]." We think that the native habitat of *E. coli* is more relevant for our case than conditions at hydrothermal vents or in earlier eras of Earth history.

Line 130: I first read this as 3% of current atmospheric CO2 levels, i.e. this is essentially always achieved. I soon realized this was wrong, but perhaps citing value of what the current atmosphere contains could help as general context

We apologize for this unclear sentence. We now corrected it: "which is equivalent to ~3% $CO_2$ in the headspace (at ambient pressure)".

Line 144-148: I'm again getting confused about some of the details of the GED shunt and its relation to the cycle… you explain how the shunt has all the reactions of the cycle except that it lacks the ability to regenerate the substrate Ru5P due to the deletion of RPE. But there is also still R5P isomerase, which of course does produce Ru5P so it's not immediately clear to me how the RPE mutant only possesses a shunt and not a cycle… Said another way it's not clear to me why RPE is critical for the operation of the GED cycle in general, and so similarly it's not clear to me that you could not in theory get the RPE mutant to grow on only CO2 if other subtle adaptation (like those you refer to elsewhere) fell into place. To be clear, I do follow how your experiments show the ability of driving Gnd to fix CO2, that is in fact quite clear and elegant, my confusing is coming in around the cycle vs shunt discussion and why you are calling something a shunt.

We thank the reviewer for raising the problem with our confusing definition of the GED shunt. Following this comment, we have considerably elaborated the explanation to make it clear what we exactly mean by this term (also see above):

"Hence, to check the feasibility of the cycle, we focused on establishing growth via the "GED shunt", representing a segment of the full cycle which consists of reductive carboxylation by Gnd and the subsequent ED pathway (blue reactions in Fig. 2A; for a similar approach see ref. [36,37]). As we show below, growth via this linear shunt requires the activity of most enzymes of the GED cycle, but relies on a pentose substrate rather than regeneration of Ru5P."

Figure 2: in the context of the previous point please carefully double check the direction of all your arrows and the names of your metabolites. Due to my confusion around the cycle vs shunt discussion I was for a while wondering whether you had swapped the R5P and Ru5P labels… Also, several arrows have different directions in Fig. 2 and 3, and shouldn't many of those reactions be bidirectional anyway? (you are showing results from different reactions that push the network in different directions in both cases).

We present the reaction arrows according to the predicted directionality of the flux they carry in the particular selection schemes shown (as indicated by Flux Balance Analysis). Indeed, in different selection strains, some of the reactions

are predicted to operate in the opposite direction. We believe that showing the actual directionality (rather than bidirectional arrows) is helpful for the reader to understand how the different biomass building blocks are generated in each strain. Following the reviewer's comment, we added a sentence in the figure legends to explain this point.

Line 163: Why do you say no growth? The right panel shows a positive slope at the beginning of the experiment even under ambient CO2. Not much perhaps, but there does appear to be some growth. This is a point that comes back in many if not most of your figures...

This is a good point indeed, which was also raised by another reviewer. Indeed, some of the strains we claim not to grow do show a slight increase in OD. (We note that our use of a log-scale makes such minute growth seem quite substantial although the actual increase in OD is very small). We are certain that this OD increase is not due to evaporation as we always use mineral oil, which, from our vast experience in these experiments, makes evaporation completely negligible even after a week or more of cultivation (while oxygen and $CO_2$ can easily penetrate it).

We emphasize that those strains that show the 'minute growth' were all constrained to less than two doublings (starting from an OD of 0.02 and ending at an OD lower than 0.08). While we cannot provide a concrete answer for why these 1-2 doublings did occur, we can speculate that it might be related to inefficient use of the GED shunt that, while operating, could not support continued growth. Indeed, in all instances where we observe a 'minute growth' phenotype, it could be potentially explained by a use of the GED shunt (e.g., all were at high $CO_2$ concentration and expressing the GED shunt enzymes).

Following the reviewer's comment, we have amended the text in all relevant instances to clarify that by 'no growth' we mean less than 2 doublings.

Line 167-168: I don't see any blue lines in Fig. 2B. I do see brown lines and upon closer inspection those appear to be the experiment in question. Again there is the point of a positive slope when you mention no growth…

Indeed a mistake from our side. We corrected the sentence to say "brown lines".

Lines 175-177: you might want to discuss your WT in the text. Partly because your observations are made against this control and so seems like they should be discussed, but I am also wondering about the observed patterns (always some labeling of one carbon, and only one carbon, in all the compounds). Do you think this is because of anaplerotic reactions in the TCA cycle causing the label to end up everywhere? This is a minor point.

In the main text, we prefer not to elaborate on the labeling within the WT strain, for the sake of shortness and readability. We feel that the substantial difference in labeling pattern between our strains and WT is enough to prove the activity of the shunt. We have, however, added a short explanatory sentence in the caption of Figure 2: "Labeling of amino acids in the WT strain stems from the natural occurrence of $^{13}C$ as well as from reactions that exchange cellular carbon with $CO_2$, e.g., the glycine cleavage system and anaplerotic/cataplerotic cycling".

Line 181: case in point about the cycle vs shunt confusion, here you delete another gene than RPE, so it is again not clear to what exactly the shunt is. It seems like perhaps you mean a group of variant pathways rather than a single thing, but it is still a bit confusing. At least in this case it is more obvious how the TKT mutant would not be able to fix CO2 without supplementation of other organics.

[end note: after completing the review and thinking it over i realized that with GED shunt you mean simply the core sequence of Gnd leading into ED glycolysis, and the cycle contains the shunt but then regenerates multiples of its inputs from the outputs. It would be good to make this really clear throughout. Similarly the confusion around whether it is correct to describe the RPE mutant as not having a complete (latent) cycle should be given some thought, as outlined above]

The reviewer is correct that the GED shunt basically refers to the core of the GED cycle, that is, the activity of Gnd and ED glycolysis. Yet, growth of our selection strains via the GED shunt also requires the activity of other GED cycle enzymes, for example, multiple glycolytic enzymes. As mentioned above, we elaborated on the explanation of the term 'shunt' in the text:

"Hence, to check the feasibility of the cycle, we focused on establishing growth via the "GED shunt", representing a segment of the full cycle which consists of reductive carboxylation by Gnd and the subsequent ED pathway (blue reactions in Fig. 2A; for a similar approach see ref. [36,37]). As we show below, growth via this linear shunt requires the activity of most enzymes of the GED cycle, but relies on a pentose substrate rather than regeneration of Ru5P."

And also, with regards to the Rpe strain:

"As mentioned above, the growth of this strain requires the simultaneous activity of most enzymes on the GED cycle, including those of glycolysis and the pentose phosphate pathway; for example, net production of erythrose 4-phosphate (E4P) from ribose requires the combined activity of Gnd, the ED pathway, and enzymes of the pentose phosphate pathway."

Line 184: it is not just the reductive oxidative pentose phosphate pathway but both the reductive and oxidative variants that are abolished in the mutant, no? Also probably better to say 'reductive' rather than 'non-oxidative'...

By 'non-oxidative' we mean the canonical pentose phosphate pathway which is neither oxidative nor reductive. The reductive pentose phosphate pathway (i.e., RuBP cycle) does not exist in *E. coli*.

Line 263-264: I again feel like the authors might want to say something about their WT control, even if just to acknowledge that the pattern is much more complex than compared to the WT that is given labeled CO2. It's just a very noticeable and elaborate pattern and so readers might appreciate some basic intuitive guide to make sense of it. But this is again a minor point.

The reviewer is indeed correct that the labeling pattern of the WT strain is interesting. However, as said before, elaborating on this pattern would make reading the manuscript quite cumbersome and we strongly prefer to avoid it as it is also not directly relevant to our story.

Line 281 and surrounding text: this CETCH acronym does not tell us much about the nature of the cycle. Perhaps the authors could say a bit more, like some of the different partial pathways it makes use of, to highlight why it should be considered 'quite complex', which without context feels a bit subjective

Following the reviewer's comments, we added sentences to explain the CETCH cycle and its complexity: "The most advanced of these pathways is the CETCH cycle [53] that combines segments of the 3-hydroxypropionate/4-hydroxybutyrate cycle [54] and the ethylmalonyl-CoA pathway [55]. The CETCH cycle was assembled *in vitro* using enzymes from nine organisms and optimized in several rounds of enzyme engineering [53, 56]."

Lines 298-299: Maybe remind the reader what this is rather than relying on acronyms that require the reader to look for it. E.g. "support growth of mutant that lacked the ability to convert X to Y, but not a mutant that lacked the ability to convert Z to Y, even though both disrupt the pentose phosphate pathway"

We prefer to be consistent with previous acronym as repeating the full enzyme names in some places but not others could be more confusing than helpful. Still, as suggested by the reviewer we added to the end of the sentence the phrase "even though both disrupt the pentose phosphate pathway".

**Reviewer #3 (Remarks to the Author)**:

In this work, the authors have demonstrated a new CO2 fixation route in E. coli by overexpressing three endogenous genes (gnd, edd, eda) and deleting one (rpe) or two (tktA and tktB) other genes. The new pathway was termed the "GED shunt". The net stoichiometry of the GED shunt is the condensation of one CO2 molecule and one pentose sugar (either ribose or xylose) to produce pyruvate and a three carbon sugar-phosphate. The operation of the GED shunt was conclusively verified using 13C tracer experiments. The authors suggest that the GED shunt is the first step towards the establishment of a "GED cycle" that could fix much more CO2.

Major concerns:

1) The GED shunt has little or no practical use, since it relies on expensive substrates (e.g. ribose) to fix a small amount of CO2, i.e. at most 17% of carbon used for cell growth or product formation is derived from fixed CO2. Other researchers have already engineered E. coli to efficiently fix CO2 using the Calvin cycle, where 100% of carbohydrates in biomass are derived from fixed CO2.

We respectfully disagree with the reviewer:

i) The GED shunt was demonstrated *in vivo* as a test to prove the *in vivo* feasibility of the GED cycle (which could support 100% of the biomass carbons originating from $CO_2$), hence our intention was to show that the combination of all key enzymes of the GED cycle can channel high enough flux to be of physiological relevance, paving the way towards future implementation of the GED cycle.

ii) Indeed, $CO_2$ fixation in *E. coli* was already demonstrated via the RuBP cycle. But it was never shown before that using the **endogenous enzymes** of heterotrophic microorganisms can support growth via a **novel pathway**. Hence, the novelty of our study does not reside in converting a heterotroph to an autotroph but rather in using the heterotroph's **own enzymes** to enable carbon fixation, thus shedding light on the emergence of new carbon fixation pathways. We now elaborate on this point in the Discussion section:

"A previous study has established the RuBP cycle in *E. coli*, demonstrating that this heterotrophic bacterium can be modified to grow autotrophically with $CO_2$ as a sole carbon source [6,7]. However, to our knowledge, the current study is the first one in which the capacity for net carbon fixation was explored *in vivo* using only endogenous enzymes of a heterotrophic host, thus shedding light on the emergence of novel carbon fixation pathways."

iii) Compared to the RuBP cycle, the GED cycle can support higher yield of both biomass and products. Following the reviewer's comments we have added a Flux Balance Analysis to quantify this:

"Indeed, using Flux Balance Analysis, we found that, compared to the RuBP cycle, the GED cycle is consistently expected to support higher yields of biomass and multiple products that are derived from pyruvate or acetyl-CoA, including ethanol, lactate, isobutanol, 2,3-butanediol, acetone, butyrate, n-butanol, citrate, itaconate, 2-ketoglutarate, and levulinic acid (Supplementary Fig. S1 and Methods)."

iv) In fact, the GED shut might have a biotechnological relevance on its own. Following the reviewer's comment, we added this analysis to the Discussion section (including a new figure and supplementary figure):

"The GED shunt might have biotechnological advantages on its own. By using Flux Balance Analysis, we found that rerouting the utilization of sugar substrates via the GED shunt is expected to increase the yield of various commercially interesting products, such as acetate, pyruvate, acetone, citrate, itaconate, and levulinic acid (Fig. 5, Supplementary Fig. S5, and Methods). This is attributed to the fact that the biosynthesis of these compounds from sugar feedstocks results in the production of excess reducing power, which the GED shunt can utilize to fix $CO_2$ and generate more product. We note that this assimilation of $CO_2$ also serves to compensate for the carbon released during the oxidation of pyruvate to acetyl-CoA, thus addressing a common challenge for the production of value-added chemicals derived from acetyl-CoA [57,58]. Further supply of reducing power by adding auxiliary substrates such as hydrogen or formate [59] can make the GED shunt advantageous over glycolysis for even more reduced products, such as ethanol, lactate, n-butanol, and isobutanol (Fig. 5, Supplementary Fig. S5, and Methods). While the RuBP shunt – a linear version of the RuBP cycle, which channels Ru5P via Rubisco – can also increase fermentative yield of some products [60-62], the GED shunt always outperforms it due to a lower ATP requirement (Fig. 5 and Supplementary Fig. S5)."

2) Throughout the manuscript, the authors suggest that the GED shunt can be eventually evolved into the cyclic GED cycle, to allow more CO2 fixation. However, there is absolutely no experimental evidence presented in this manuscript that demonstrates that this can be achieved with the current strains. In fact, careful inspection of the reaction required for the GED cycle suggests that the described strains used for GED shunt can never be used for the GED cycle, since the GED-shunt strains are missing key enzymes (i.e. rpe and tktAB) that are required for the operation of the GED cycle. Thus, contrary to the suggestion of the authors, the strains reported in this work cannot be evolved to establish the GED cycle. This greatly diminishes the significance of the present study. It is essentially a dead-end from a pathway engineering perspective.

Following the reviewer's comment, we added a full analysis of a new selection strain that, while still allowing selection for the activity of the GED shunt (i.e., preventing the utilization of a pentose substrate as carbon source in a GED shunt-independent manner) can be evolved to utilize the full GED cycle. We describe this in a new section in the Results, where we also explain in detail the benefits of this strain over the previous ones:

"While the Δ*rpe* and Δ*tktAB* strains were useful selection platforms to test the activity of the GED shunt, they are, in a sense, 'metabolic dead-ends'. This is because the activities of both ribulose-phosphate 3-epimerase (Rpe) and transketolase (Tkt) are essential for the operation of the full GED cycle, that is, for the regeneration of Ru5P from GAP. To address this problem, we aimed to construct a strain which keeps all necessary enzymes of the GED cycle intact, while still allowing to select for the activity of the GED shunt, i.e., preventing utilization of a pentose substrate as sole carbon source via the canonical pentose phosphate pathway. Such a strain would enable a smooth transition from GED shunt-dependent growth on a pentose substrate towards autotrophic growth via the GED cycle.

We therefore constructed a strain deleted in all enzymes that can metabolize fructose 6-phosphate, directly or indirectly, into a downstream glycolytic intermediate (Δ*pfkAB* Δ*fsaAB* Δ*fruK*) or channel it into the oxidative pentose phosphate pathway (Δ*zwf*). The latter gene deletion should also support the activity of the GED shunt, as was shown above within the Δ*tktAB* context. The strain containing all of these deletions, which we term ΔPZF, establishes a uni-directional block within the pentose phosphate pathway. That is, growth on a pentose substrate is not possible due to the accumulation of fructose 6-phosphate that prevents further conversion of pentose phosphates into GAP (Fig. 4A) [7]. In contrast, flux in the opposite direction, as required for the GED cycle, can still occur, since fructose 1,6-bisphosphate can be dephosphorylated to fructose 6-phosphate which is then used to regenerate Ru5P.

To establish growth of the ΔPZF strain on xylose via the GED shunt, we overexpressed Gnd, Edd, and Eda. However, transforming the ΔPZF strain with pGED failed to support growth on xylose even at elevated $CO_2$ (less than two doublings, red line in Fig. 4B). Hence, we again harnessed natural selection and performed short-term evolution by incubating the strain in multiple test-tubes for an extended period of time in xylose minimal medium at 37°C and 20% $CO_2$. Within 6-8 days, three parallel cultures started growing. Isolated clones from two of these three mutant cultures displayed a fairly high growth rate (doubling time of 8-10 hours, green and blue lines in Fig. 4B), while clones from the third culture showed considerably slower growth (doubling time > 50 h, orange line in Fig. 4B). We conducted $^{13}$C-labeling experiments using the fastest-growing strain (ΔPZF +pGED mutant "C") and found that it displayed a labeling pattern almost identical to that of the Δ*tktAB* Δ*zwf* +pGED strain described above, thus confirming growth via the GED shunt (Fig. 4C and Supplementary Fig. S4). We sequenced the genomes of the mutant strains, compared them to the parental strain, and discovered several mutations (Supplementary Table 1). All isolated colonies from the two fast-growing cultures shared an identical mutation at the start of an L-leucyl-tRNA (*leuX*) and, in most colonies, *avtA*, encoding for valine-pyruvate aminotransferase, had mutated. While the exact contribution of these mutations to the growth phenotype remains elusive and could be further investigated, the isolated strains provide a promising starting point for evolution of the full GED cycle."

We also refer to this point and possible future evolution in the Discussion section:

"Importantly, the establishment of the RuBP cycle in *E. coli* required long-term adaptive evolution of the microbe under selective conditions, which modulated the partitioning of metabolic fluxes between carbon fixation and biosynthetic pathways [23,63]. We expect that autotrophic growth via the GED cycle can be achieved in a similar manner. The ΔPZF strain serves as an ideal starting point for such a future evolution experiment, as its growth is dependent on the activity of the GED shunt while it still harbors all necessary enzymes to run the GED cycle. The gradual evolution of autotrophic growth via the GED cycle would be achieved via the additional expression of formate dehydrogenase as an energy-supplying module and long-term cultivation with limiting amounts of xylose and saturating amounts of $CO_2$ and formate [6,7]."

Reviewer #1 (Remarks to the Author):

The authors have addressed most of the reviewers' comments and present a much improved version of their manuscript. However, there are two areas in which the manuscript can be further improved:

1- Flux balance analysis (FBA):
- The section on "Yield estimation via Flux Balance Analysis" should be modified or removed altogether. There are questionable assumptions such as "We further removed the ATP maintenance reaction (ATPM) due to the fact that, rather than estimating growth rate, we used FBA to estimate the maximal yield. We used the model with these modifications as a ‘wild-type’ reference." If ATP is not taken into account and the objective function is product yield, why to conduct FBA? This is simulating a scenario that will never exist in vivo. If the authors desire to simulate product synthesis in vivo, they could do so for growing or non-growing cultures.
- The authors make very strong claims based on their FBA, including about the superiority of the GED cycle over the RuBP cycle based on biomass product yields obtained from the FBA. However, the differences are very small and such strong claims should be backed by a more detailed analysis, such as flux variability analysis. Also, as mentioned above, the simulations that calculate product yields are flawed.
- There is no need to conduct FBA to claim that a CO2-utilizing pathway can improve the yield of oxidized products derived from glucose by recycling the CO2 generated by certain glycolytic pathways and pyruvate dissimilation via PDHC. This is well known and the issue has been addressed before, so citing relevant literature should suffice (Front. Bioeng. Biotechnol., 10 January 2020 | https://doi.org/10.3389/fbioe.2019.00446; Proc. Natl. Acad. Sci. U.S.A. 115, 3538–3546. doi: 10.1073/pnas.1802191115; Nature 502, 693–697. doi: 10.1038/nature12575).

2- Growth via the GED shunt in a strain that could support cyclic flux
- This is a new section that was added in response to a strong criticism from one of the reviewers. This is good data and analysis, but somewhat inconclusive as the role of the identified mutations was not established. The authors state that "While the exact contribution of these mutations to the growth phenotype remains elusive and could be further investigated, the isolated strains provide a promising starting point for evolution of the full GED cycle". I think the authors should at least provide a strong hypothesis about how the identified mutation(s) support the observed phenotype.

The authors should also avoid making explicit claims of superiority and novelty, as this often detracts from the quality of the work (which is high) and is unnecessary are sometimes inaccurate. For example, see above for claims of superiority of GED cycle over the RuBP cycle. Similarly, the use of native enzymes/combination of native enzymes to achieve a non-native function has been widely reported in the literature, as it is the repurposing of enzymes (including reversibility) for non-native functions.

Reviewer #2 (Remarks to the Author):

The authors have done a great job addressing all my comments and i have nothing further to add. Congratulations on a nice paper.

Rogier Braakman

Reviewer #3 (Remarks to the Author):

The authors have addressed most of my concerns. I agree that the new strain (i.e. PZF) is a much better starting strain for adaptive evolution of the GED cycle compared to the rpe-KO and tktAB-KO strains, which were metabolic dead-ends. One concern is that the authors now assume that they have

eliminated all pathways for fructose-6-phosphate (F6P) conversion. However, one recent study (Metab Eng, 52: 168-177, 2019) used a similar strain (where pfkA and zwf were both deleted) and showed that xylose (via F6P) was converted to glucose. The authors should comment how this additional pathway that they have not considered can negatively impact their evolution strategy. I suggest that the authors test if their PZF-strain also converts xylose into glucose.

**RESPONSE TO REVIEWERS COMMENTS**

**Reviewer #1 (Remarks to the Author):**

The authors have addressed most of the reviewers' comments and present a much improved version of their manuscript.

We thank the reviewer for the support.

However, there are two areas in which the manuscript can be further improved:

1- Flux balance analysis (FBA):

- The section on "Yield estimation via Flux Balance Analysis" should be modified or removed altogether. There are questionable assumptions such as "We further removed the ATP maintenance reaction (ATPM) due to the fact that, rather than estimating growth rate, we used FBA to estimate the maximal yield. We used the model with these modifications as a 'wild-type' reference." If ATP is not taken into account and the objective function is product yield, why to conduct FBA? This is simulating a scenario that will never exist in vivo. If the authors desire to simulate product synthesis in vivo, they could do so for growing or non-growing cultures.

We agree with the reviewer that our use of FBA was somewhat uncommon since we used it merely as a tool to conduct a purely stoichiometric analysis and estimate maximum theoretical yields without considering (non-growth-associated) maintenance costs. This form of analysis allowed us to avoid assumptions about sugar uptake rates in the different simulated conditions. Yet, as the reviewer noted, the removal of the maintenance reaction represents an unrealistic estimation of *in vivo* yields. Thus, as suggested by the reviewer, we changed the analysis to include the ATPM reaction, i.e. to take into account the current estimate for non-growth-associated maintenance (from the model *i*ML1515). Since this strictly requires an assumption on feedstock uptake rates, we have set these to experimentally determined values for xylose and glucose uptake, as reported in the literature.

The following changes have been made in the manuscript:

1. Figure 5B and Supplementary Figure S4 were updated with the new results from the flux-balance analysis including ATPM.

2. The following sentences were corrected/added in the corresponding methods section (Yield estimation via Flux Balance Analysis):

   "*The default value of the model for the non-growth-associated ATP maintenance reaction was used (ATPM; 6.86 mmol/gDW/h), to simulate maximal theoretical product yields in stationary/non-growing cells.*"

   "*The uptake rates for xylose and glucose were set to experimentally determined values for anaerobic, fermentatively growing E. coli cultures (xylose: 10.8 mmol/gDW/h; glucose: 13.1 mmol/gDW/h)* [78]*.*"

3. We now clarify this point also in the discussion section:

   "Indeed, we applied Flux Balance Analysis to simulate production in non-growing cells (Methods) and found that …"

- The authors make very strong claims based on their FBA, including about the superiority of the GED cycle over the RuBP cycle based on biomass product yields obtained from the FBA. However, the differences are very small and such strong claims should be backed by a more detailed

analysis, such as flux variability analysis. Also, as mentioned above, the simulations that calculate product yields are flawed.

We agree with the reviewer that our comparison of the GED cycle and the RuBP cycle for the use of $CO_2$ as sole carbon source showed rather minor differences in yield. Thus, we have removed Supplementary Figure 1 (comparing biomass/product yields of the GED cycle vs. the RuBP cycle on $CO_2$). We further deleted the corresponding results section with strong statements on energetic superiority of the GED cycle.

- There is no need to conduct FBA to claim that a CO2-utilizing pathway can improve the yield of oxidized products derived from glucose by recycling the CO2 generated by certain glycolytic pathways and pyruvate dissimilation via PDHC. This is well known and the issue has been addressed before, so citing relevant literature should suffice (Front. Bioeng. Biotechnol., 10 January 2020 | https://doi.org/10.3389/fbioe.2019.00446; Proc. Natl. Acad. Sci. U.S.A. 115, 3538–3546. doi: 10.1073/pnas.1802191115; Nature 502, 693–697. doi: 10.1038/nature12575).

We agree with the reviewer that previous studies have shown that co-utilization of $CO_2$ can increase yields of certain products. Indeed, we include multiple references demonstrating this point. Following the reviewer comment, we adjusted the text in the Discussion section to put more emphasis on these previous works. Importantly, however, our main focus is to show how the GED shunt compares to the RuBP shunt, a more established option for the co-utilization of $CO_2$. This comparison shows that the GED shunt is consistently predicted to achieve higher yields. This is a non-trivial result. The corresponding discussion paragraph now reads as follows:

"The GED shunt might have biotechnological applications on its own. Previous studies have demonstrated that co-assimilation of $CO_2$ can increase bio-production yields from common feedstocks such as sugars [57-59]. This is attributed to the fact that the biosynthesis of certain compounds from sugars results in the production of excess reducing power, which can be utilized to fix $CO_2$ and thereby generate more product. Such assimilation of $CO_2$ can also serve to compensate for the carbon released during the oxidation of pyruvate to acetyl-CoA, thus addressing a common challenge for the production of value-added chemicals derived from acetyl-CoA [60-62]. Indeed, we applied Flux Balance Analysis to simulate production in non-growing cells (Methods) and found that rerouting the utilization of sugar substrates via the GED shunt is expected to increase the yield of various commercially interesting products, such as acetate, pyruvate, acetone, citrate, and itaconate (Fig. 5, Supplementary Fig. S4). Further supply of reducing power by adding auxiliary substrates such as hydrogen or formate [63] can make the GED shunt advantageous over glycolysis for even more reduced products, such as ethanol, lactate, 1-butanol, and fatty acids (Fig. 5, Supplementary Fig. S4). While the RuBP shunt – a linear version of the RuBP cycle, which channels Ru5P via Rubisco – can also increase fermentative yield of some products [57-59], the GED shunt always outperforms it due to a lower ATP requirement (Fig. 5 and Supplementary Fig. S4)."

2- Growth via the GED shunt in a strain that could support cyclic flux

- This is a new section that was added in response to a strong criticism from one of the reviewers. This is good data and analysis, but somewhat inconclusive as the role of the identified mutations was not established. The authors state that "While the exact contribution of these mutations to the growth phenotype remains elusive and could be further investigated, the isolated strains provide a promising starting point for evolution of the full GED cycle". I think the authors should at least provide a strong hypothesis about how the identified mutation(s) support the observed phenotype.

The reviewer is correct that we have provided no speculation on the causal link between the identified mutations and the phenotype. This is due to the fact that the key mutations which distinguish the evolved strains from the parental strain are a SNP in a leucyl-tRNA (leuX) or a frameshift in RNase E. These components are involved in a multitude of cellular processes.

Identifying the specific causal relationship of these mutations to the GED-dependent growth phenotype would be very difficult, as these mutations are likely to cause quite extensive changes in cellular physiology. Despite extensive literature search, we can at present offer no mechanistic prediction of the exact effect of the leucyl-tRNA mutation on its function. We also have not found a plausible link between RNase E and leuX, which may have pointed to a common mechanism of these mutations which arose in independent lineages. We note that several recent high-impact studies do not elucidate the specific contribution of such pleiotropic mutations identified after adaptive evolution, presumably for similar reasons of infeasibility. We would thus prefer not to speculate in the main text and have instead added our current hypothesis in Supplementary Table 1 (see below). In our opinion, confirming this hypothesis is beyond the scope of this study, which focuses on demonstrating the *in vivo* feasibility of the GED cycle. The text we added to Supplementary Table 1:

*"The identified mutation in the L-leucyl-tRNA (leuX) was found in all mutant clones from the independent evolution cultures "B" and "C", which display considerably faster growth than mutants from culture "A". The mutation is a single-nucleotide polymorphism located at the second base from the 5'-end of the mature tRNA, outside its anticodon loop. We hypothesize that this mutation negatively affects maturation and/or folding of the tRNA and thereby modifies translation rates of any gene containing UUG-codons (13% of leucine-encoding codons), although other leucyl-tRNAs are known to compensate partially for loss-of-function or deletion of leuX (FEBS Letters 344 (1994) 31-34; J. Mol. Biol. (1979) 129, 567-585). Notably, the* edd *gene (encoding 6-phosphogluconate dehydratase, one of the key enzymes in the GED pathway) contains 12 codons normally recognized by leuX. Therefore it is possible that a loss-of-function in leuX optimized expression of* edd *at the translational level, possibly by reducing its protein abundance or by improving* edd *maturation (e.g. incorporation of the required iron-sulfur cluster) via slowed translation."*

The authors should also avoid making explicit claims of superiority and novelty, as this often detracts from the quality of the work (which is high) and is unnecessary are sometimes inaccurate. For example, see above for claims of superiority of GED cycle over the RuBP cycle. Similarly, the use of native enzymes/combination of native enzymes to achieve a non-native function has been widely reported in the literature, as it is the repurposing of enzymes (including reversibility) for non-native functions.

We appreciate the note that fewer explicit claims of superiority and novelty would improve the quality of the manuscript. We therefore screened the text for such instances and removed them. As suggested, we have also removed the strong claims on energetic superiority of the GED cycle (see above).

**Reviewer #2 (Remarks to the Author):**

The authors have done a great job addressing all my comments and i have nothing further to add. Congratulations on a nice paper.

Rogier Braakman

We thank the reviewer for his support.

**Reviewer #3 (Remarks to the Author):**

The authors have addressed most of my concerns. I agree that the new strain (i.e. PZF) is a much better starting strain for adaptive evolution of the GED cycle compared to the rpe-KO and tktAB-KO strains, which were metabolic dead-ends. One concern is that the authors now assume that they have eliminated all pathways for fructose-6-phosphate (F6P) conversion. However, one recent study (Metab Eng, 52: 168-177, 2019) used a similar strain (where pfkA and zwf were both deleted)

and showed that xylose (via F6P) was converted to glucose. The authors should comment how this additional pathway that they have not considered can negatively impact their evolution strategy. I suggest that the authors test if their PZF-strain also converts xylose into glucose.

We thank the reviewer for the suggestion. We have performed an assay to detect the presence of glucose in the supernatants of corresponding mutant strains. We could not identify glucose in any sample. We have added the following paragraph to the Results section:

"*A recent study reported that a similar E.coli deletion strain (deleted in pfkA, zwf, and the glucose uptake system) was able to grow on a xylose minimal medium, but was accompanied by the secretion of a substantial amount of glucose (34% of consumed xylose)* [50]*. Such secretion of a dephosphorylated sugar could relieve the inhibitory accumulation of F6P and thus theoretically enable the growth of the ΔPZF strain even without the activity of the GED shunt. However, the growth of the ΔPZF mutants we identified cannot be explained by such a phenomenon since: (i) growth at ambient* $CO_2$ *was not observed (Figure 4B), confirming strict dependency on the activity of the GED shunt; and (ii) no glucose could be detected in the supernatants of the growing cells (Methods). This excludes the possibility that growth was even partially supported by conversion of xylose into glucose*."

The following section has been added to the Methods:

"***Determination of extracellular glucose concentrations via enzymatic assay.*** *Glucose concentrations in supernatants of ΔPZF mutant cultures were determined using a commercial glucose oxidase-based assay kit following the manufacturer's instructions (Merck, Darmstadt, Germany; Catalogue No. GAG020). In brief, three independent cultures each of ΔPZF mutant B1 and C1 were grown in 3 mL M9 medium with 20 mM xylose, and samples were taken in exponential phase (*$OD_{600}$ *= 0.4-0.8) and in early stationary phase (*$OD_{600}$ *~ 1.1), centrifuged for 3 min at 20.000 g and supernatants frozen at -20°C for later use. A standard curve with varying xylose concentrations confirmed negligible background signal from the xylose contained in the medium (20 mM xylose resulting in a signal corresponding to 0.163 mM (i.e. 0.029 mg/mL) glucose). The lower detection limit for glucose in the supernatant was thus assumed to be 0.17 mM, i.e. such concentrations or higher would be detected even in the case of complete consumption of all xylose in the media by the growing cells (i.e. an order of magnitude below relevant reported values for glucose excretion: 1.8 mM per unit increase in* $OD_{600}$ [50]*). Glucose standards were prepared in triplicate in the growth medium at the following concentrations (mg/mL): 0, 0.02, 0.04, 0.08. Assays were performed by mixing 400 µL of standard or supernatant sample with 800 µL reagent mix, prepared following the manufacturer's instructions, and incubated at 37°C for 30 min. The reaction was stopped by adding 800 µL of sulfuric acid (6M). Absorbance was measured at 540 nm and sample glucose concentrations determined by means of a standard curve. No glucose signal above background was detected in any culture sample*."

Regarding the reviewer's comment about the possible emergence of a glucose-secretion phenotype in the evolution of the full cycle, we note that we describe and discuss a chemostat-based evolution strategy with limiting amounts of a pentose feedstock. Thus, we presume that the secretion of glucose or other organic compounds would be highly wasteful and we do not expect it to confer a growth advantage to the corresponding mutant cells.

Reviewer #1 (Remarks to the Author):

The authors have satisfactorily addressed reviewers' comments.

Reviewer #3 (Remarks to the Author):

The authors have addressed my concerns.
